# A spiking Basal Ganglia model of synchrony, exploration and decision making

**DOI:** 10.3389/fnins.2015.00191

**Published:** 2015-05-27

**Authors:** Alekhya Mandali, Maithreye Rengaswamy, V. Srinivasa Chakravarthy, Ahmed A. Moustafa

**Affiliations:** ^1^Computational Neuroscience Lab, Department of Biotechnology, Bhupat and Mehta School of BioSciences, Indian Institute of Technology MadrasChennai, India; ^2^Marcs Institute for Brain and Behaviour and School of Social Sciences and Psychology, University of Western SydneySydney, NSW, Australia

**Keywords:** Basal Ganglia, Izhikevich neurons, synchronization, n-arm bandit task, exploration

## Abstract

To make an optimal decision we need to weigh all the available options, compare them with the current goal, and choose the most rewarding one. Depending on the situation an optimal decision could be to either “explore” or “exploit” or “not to take any action” for which the Basal Ganglia (BG) is considered to be a key neural substrate. In an attempt to expand this classical picture of BG function, we had earlier hypothesized that the Indirect Pathway (IP) of the BG could be the subcortical substrate for exploration. In this study we build a spiking network model to relate exploration to synchrony levels in the BG (which are a neural marker for tremor in Parkinson's disease). Key BG nuclei such as the Sub Thalamic Nucleus (STN), Globus Pallidus externus (GPe) and Globus Pallidus internus (GPi) were modeled as Izhikevich spiking neurons whereas the Striatal output was modeled as Poisson spikes. The model is cast in reinforcement learning framework with the dopamine signal representing reward prediction error. We apply the model to two decision making tasks: a binary action selection task (similar to one used by Humphries et al., 2006) and an n-armed bandit task (Bourdaud et al., 2008). The model shows that exploration levels could be controlled by STN's lateral connection strength which also influenced the synchrony levels in the STN-GPe circuit. An increase in STN's lateral strength led to a decrease in exploration which can be thought as the possible explanation for reduced exploratory levels in Parkinson's patients. Our simulations also show that on complete removal of IP, the model exhibits only Go and No-Go behaviors, thereby demonstrating the crucial role of IP in exploration. Our model provides a unified account for synchronization, action section, and explorative behavior.

## Introduction

Imagine a situation where you would like to dine out and are in search of suitable restaurants. Some restaurants you know for sure are good, and others you have no idea about. In other words you have two fundamentally different options of which one is to order your favorite dish and play it safe (i.e., “exploit”) while the other is to try something new (i.e., “explore”). Further, an unexpected weather change would force you to stay at home (i.e., a No Go decision). How does our brain make a decision in such a scenario? Depending on the situation, an optimal decision could be to either explore, exploit or to take no action (Cohen et al., 2007; Prescott et al., 2007).

A group of subcortical structures collectively called the Basal ganglia (BG) play an important role in many cognitive processes (Gurney et al., 2001a,b; Humphries and Gurney, 2002; Chakravarthy et al., 2010; Schroll et al., 2012; Yucelgen et al., 2012; Chersi et al., 2013) including decision making and action selection. The BG circuit includes the neo-striatum (caudate and putamen), Globus pallidus (externa, GPe, and interna, GPi), subthalamic nucleus (STN), and substantia nigra (pars compacta, SNc and pars reticulata, SNr). BG receive inputs from the cortex through the striatum and STN (Maurice et al., 1998; Aravamuthan et al., 2007) and project through SNr and GPi, the output nuclei of BG, via thalamus (Albin et al., 1989) to motor and executive areas of the cortex (Steiner and Tseng, 2010). Classically BG pathways are segregated into the indirect pathway (IP) constituting a part of the striatum, GPe and STN projecting to GPi (Gerfen and Surmeier, 2011) and the direct pathway (DP) constituting the projection from the striatum to GPi (Gerfen and Surmeier, 2011). The final “action selection” is based on the combined contributions of the two pathways at output nuclei (Smith et al., 1998). The effect of dopamine (DA) on BG pathways and decision making has been well known (Rogers, 2010). Under low DA conditions, IP is more active than DP leading to “No-Go” behavior (Frank, 2005) whereas in high DA conditions DP is more active than IP leading to “Go” (Chevalier and Deniau, 1990). But this traditional explanation of action selection in binary terms of Go/No Go misses out “exploration” and its possible neural substrates out of the picture.

The ability to switch between explorative and exploitative behavior during decision making drew the attention of neuroscientists to study and characterize the corresponding anatomical substrates. It has been suggested that the pallidum, in its interactions with the noradrenergic system, controls the balance between exploration-exploitation (Russell et al., 1992; Aston-Jones et al., 1994; Doya, 2002). Humphries et al. (2007) argue that the brainstem specifically medial reticular formation (mRF) might be the substrate for action selection (Humphries et al., 2007). Schroll et al. (2012) presented a model of working memory sub-served by the cortico-basal-ganglia-thalamic loops where exploration in the model was obtained by the addition of noise to neural dynamics, but no anatomical substrate was suggested (Schroll et al., 2012). Chersi et al. (2013) simulate the role of BG and prefrontal cortex in goal-oriented learning vs. habitual learning and hypothesize that exploration emerges during the “up” state of striatal neurons (Chersi et al., 2013). Shouno et al. (2009) built a spiking network model of BG where the IP selects an action and the DP determines the timing of the selected action. Though the model was able to show exploration in terms of variability in action selection, there was no component of learning in the network (Shouno et al., 2009). Stewart et al. (2012) simulated the rat bandit task experiment using leaky integrate and fire model of cortex and BG where the spiking activity of ventral striatum during a response was measured. Though the model showed behavioral learning, anatomical substrate for exploration was not suggested (Stewart et al., 2012). A recent study by Humphries et al. (2012) suggest the role of tonic DA in setting the exploitation- exploration tradeoff (Humphries et al., 2012) in basal ganglia. Among computational models of BG, very few simulated the neural substrates for exploration within the BG system. The study by Archibald et al. (2013a) on PD patients indicates a decrease in exploration behavior compared to healthy controls during a visuo-spatial task (Archibald et al., 2013a,b).

Chakravarthy et al. (2010) suggested that STN-GPe loop, a coupled excitatory-inhibitory network in the IP, might be the substrate for exploration (Chakravarthy et al., 2010). It is well known that coupled excitatory-inhibitory pools of neurons can exhibit rich dynamic behavior like oscillations and chaos (Borisyuk et al., 1995; Sinha, 1999). This hypothesis has inspired models simulating various BG functions ranging from action selection in continuous spaces (Krishnan et al., 2011), reaching movements (Magdoom et al., 2011), spatial navigation (Sukumar et al., 2012), precision grip (Gupta et al., 2013), and gait (Muralidharan et al., 2013) in normal and Parkinsonian conditions. Using a network of rate-coding neurons, Kalva et al. (2012) showed that exploration emerges out of the chaotic dynamics of the STN-GPe system (Kalva et al., 2012). Most rate coded models, by design, fail to capture dynamic phenomena like synchronization found in more realistic spiking neuron models (Terman et al., 2002; Park et al., 2010, 2011). Synchronization within BG nuclei had gained attention since the discovery that STN, GPe, and GPi neurons show high levels of synchrony in Parkinsonian conditions (Bergman et al., 1994; Bevan et al., 2002; Hammond et al., 2007; Tachibana et al., 2011; Weinberger and Dostrovsky, 2011). This oscillatory activity was found to be present in two frequency bands, one around the tremor frequency [2–4 Hz] and another in [10–30 Hz] frequency (Weinberger and Dostrovsky, 2011). Park et al. (2011) report the presence of intermittent synchrony between STN neurons and its Local field potentials (LFP), recorded using multiunit activity electrodes from PD patients undergoing Deep Brain Stimulation (DBS) surgery (Park et al., 2011) which is absent in healthy controls.

One of the key objectives of the current study is to use a 2D spiking neuron model to understand and correlate STN-GPe's synchrony levels to exploration. As the second objective, we apply the above-mentioned model to the n-armed bandit problem of Daw et al. (2006) and Bourdaud et al. (2008) with the specific aim of studying the contributions of STN-GPe dynamics to exploration. The proposed model shares some aspects of classical RL-based approach to BG modeling. For example, dopamine signal is compared to reward prediction error (Schultz, 1998). Furthermore, DA is allowed to control cortico-striatal plasticity [47], modulate the gains of striatal neurons (Kliem et al., 2007; Hadipour-Niktarash et al., 2012) and influence the dynamics of STN-GPe by modulating the connections (Kreiss et al., 1997; Fan et al., 2012).

The paper is organized as follows. Section Methods: Model Details describes the model architecture and equations used in the simulations. Section Results presents the results. Implications of the modeling study are discussed in the final section.

## Methods: model details

**Figure 1:**
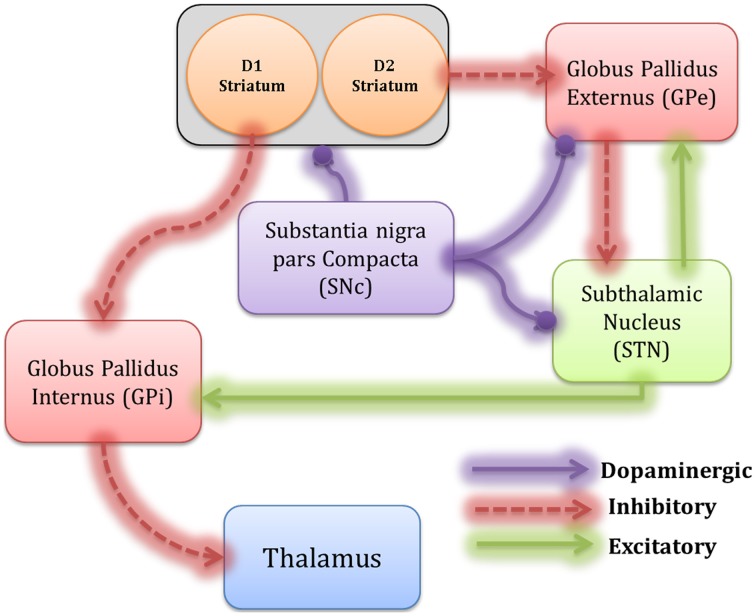
**The architecture of the proposed spiking neural Basal Ganglia model, which includes Striatum, GPe, GPi, STN, thalamus, and SNc.** The inhibitory connections are represented by dotted lines and the excitatory connections by solid lines. The modulatory effect of DA is shown by the solid line with a ball.

The model consists of the striatum, STN, GPe, GPi, and SNc (Figure 1). Modeling details of various BG nuclei are described below. All the simulations were coded using MATLAB v2012.

### Striatum

Striatal neurons display irregular firing patterns during wakeful stage (Stern et al., 1998; Mahon et al., 2006) which was accounted by modeling the striatal (both D1 and D2) output as Poisson process. The presynaptic potentials due to this striatal output [D1R expressing and D2R expressing medium spiny neurons (MSNs), (Kreitzer, 2009)] was represented as 2 unconnected pools (50 × 50 each) that give rise to GABAergic current from D1 striatum to GPi (Gerfen et al., 1990; Gurney et al., 2001a; Gerfen and Surmeier, 2011) and D2 striatum to GPe neurons (Gerfen et al., 1990) respectively.

### Izhikevich neuron model

Izhikevich spiking neuron models have an advantage of being computationally inexpensive compared to biophysical models yet capable to capture various neuronal properties such as firing rate and spike pattern (Izhikevich, 2003) which is absent in rate coded models. The key modules in BG circuit including GPe, STN, and GPi (Figure 1) were modeled using Izhikevich neuron models arranged in a 2D lattice (= 50 × 50) consisting of 2500 neurons each. The Izhikevich parameters (a, b, c, d) for STN neuron were adapted from (Michmizos and Nikita, 2011), GPe and GPi neurons were modeled as tonically spiking Izhikevich neurons (Izhikevich, 2003). The external current (I^x^) was adjusted to match the published firing frequencies of these neuronal types (Modolo et al., 2007). The values of the Izhikevich parameters are given in Table 1.

(1) $$\frac{dv_{ij}^{x}}{dt}=0.04{\left(v_{ij}^{x}\right)}^{2}+5v_{ij}^{x}-u_{ij}^{x}+140+I_{ij}^{x}+I_{ij}^{syn}$$

(2) $$\frac{du_{ij}^{x}}{dt}=a(bv_{ij}^{x}-u_{ij}^{x})$$

(3) $$if\;v_{ij}^{x}\geq v_{peak}\left\{\begin{array}{l} v_{ij}^{x}\leftarrow c\;\\ u_{ij}^{x}\leftarrow u_{ij}^{x}+d \end{array}\right\}$$

where, *v*^*x*^_*ij*_ = membrane potential, *u*^*x*^_*ij*_ = membrane recovery variable, *I*^*Syn*^_*ij*_ = total synaptic current received, *I*^*x*^_*ij*_ = external current applied to neuron x at location (*i*, *j*), *v*_*peak*_ = maximum voltage set to neuron (+30 mv) with x being STN or *GPe* or *GPi* neuron.

**Table 1:** **Gives the values and the description of the parameters used in the model and simulation**.

**Parameter**	**Values with description**			
	**STN**		**GPe**	**GPi**
Izhikevich parameters	*a* = 0.005, *b* = 0.265, *c* = −65, *d* = 1.5		*a* = 0.1, *b* = 0.2, *c* = −65, *d* = 2	*a* = 0.1, *b* = 0.2, *c* = −65, *d* = 2
External current (I)	I_STN_ = 30		I_GPe_ = 10	I_GPi_ = 10
StrD1→GPi	0.8		Synaptic weight between D1 striatum and GPi	
W_StrD2→GPe_	1		Synaptic weight between D2 striatum and GPe	
DA	0.1–0.9 in increments of 0.1			
A_D1_	10	Amplitude of GABAergic current from D1 striatum to GPi neurons due to DA		
A_D2_	7.5	Amplitude of GABAergic current from D2 striatum to GPe neurons due to DA		
λ^Str^	7.5	Slope of the Gain functions (cD1 and cD2)		
Mg^2+^	1 nM	Concentration of Magnesium ions in nM		
E_AMPA_	0 mv	Synaptic potential of AMPA receptor-associated channel		
E_NMDA_	0 mV	Synaptic potential of NMDA receptor-associated channel		
E_GABA_	−60 mV	Synaptic potential of GABA receptor-associated channel		
w_sg_	1	Synaptic weight for excitatory STN to GPe projection		
w_gs_	20	Synaptic weight for inhibitory GPe to STN projection		
cd2	0.1	Parameter that affects the STN→GPe (w_sg_) and GPe→STN(w_gs_) weights		
τ_AMPA_	6 ms	Time decay constant for AMPA receptor		
τ_NMDA_	160 ms	Time decay constant for NMDA receptor		
τ_GABA_	4 ms	Time decay constant for GABA receptor		
τ_NMDA_GPi_	67 ms	Time decay constant for NMDA receptor of GPi neurons		
r_s_	1	Radius of STN laterals Gaussian		
r_g_	0.5	Radius of GPe laterals Gaussian		
cD21	0.1	Parameter that affects the radius of STN and GPe laterals		
A_GPe_	1	Synaptic strength within GPe laterals		
A_STN_	0.2	Synaptic strength within STN laterals		
nlat_STN−_	5	# of lateral connections considered in STN neurons		
nlat_GPe−_	11	# of lateral connections considered in GPe neurons		
w_*STN*→GPi_	1.15	Synaptic weight between STN and GPi		

STN, Sub Thalamic Nucleus; GPe, Globus Pallidus Externa; GPi, Globus Pallidus Interna.

#### Synaptic connections

The synaptic connectivity between the nuclei was considered as one to one as in Dovzhenok and Rubchinsky (2012) and was modeled as (similar to Humphries et al., 2009)

(4) $$\tau_{\text{Recep}}*\frac{dh_{ij}^{x\to y}}{dt}=-h_{ij}^{x\to y}(t)+S_{ij}^{x}(t)$$

(5) $$I_{ij}^{x\to y}(t)=W_{x\to y}*h_{ij}^{x\to y}(t)*(E_{\text{Recep}}-V_{ij}^{y}(t))$$

The effect of voltage-dependent magnesium channel on NMDA current (Jahr and Stevens, 1990) was modeled as,

(6) $$B_{ij}(v_{ij})=\frac{1}{1+(\frac{Mg^{2+}}{3.57}*e^{-0.062*V_{ij}^{y}(t)})}$$

where, τ_*Recep*_ = decay constant for synaptic receptor, *E*_*Recep*_ = receptor associated synaptic potential (Recep = AMPA/GABA/NMDA), S^*x*^_*ij*_ = Spiking activity of neuron “*x*” at time “*t*,” h^*x*→*y*^_*ij*_ = gating variable for the synaptic current from “*x*” to “*y*,” *W*_*x*→*y*_ = synaptic weight from neuron “*x*” to “y,” *Mg*^2+^ = Magnesium ion concentration and *V*^*y*^_*ij*_ = membrane potential of the neuron “*y*” for the neuron at the location (*i*, *j*) The time constants of GABA, AMPA, and NMDA in STN and *GPe* were chosen from (Götz et al., 1997) are given in Table 1. All the synaptic connections with their respective variables are described in Table 2 and the values of parameters are given in Table 1.

**Table 2:** **Gives a description of all the synaptic variables of various synaptic currents modeled using Equations (4) and (8) in Section Synaptic Connections**.

**Variable**	**Description**
*h*^*StrD*1*x*→*GPi*^_*ij*_	Gating variables for GPi neuron due to GABAergic projections from D1 striatum. “*x*” represents the input #. For example, if there are 2 inputs presented to the model, *x* = 1, 2
*h*^*StrD*2*x*→*GPe*^_*ij*_	Gating variable for GPe neuron due to GABAergic projections from D2 striatum
*h*^*AMPA→GPe*^_*ij*_/*h*^*NMDA→GPe*^_*ij*_	Gating variable for GPe neuron due to glutamatergic input from STN due to either NMDA or AMPA receptor
*h*^*GABA→STN*^_*ij*_	Gating variable for STN neuron due to GABAergic input from GPe neuron
*h*^*AMPA→STN*^_*ij*_/*h*^*NMDA→STN*^_*ij*_	Gating variable for STN neuron due to glutamatergic input from its collaterals due to either NMDA or AMPA receptor
*h*^*GABA→GPe*^_*ij*_	Gating variable for GPe neuron due to GABAergic input from its collaterals.
*I*^*StrD*1→*GPi*^_*ij*_	Inhibitory GABAergic current to GPi neuron from D1 striatum
*I*^*StrD*2→*GPe*^_*ij*_	Inhibitory GABAergic current to GPe neuron from D2 striatum
*I*^*AMPA→GPe*^_*ij*_/*I*^*NMDA→GPe*^_*ij*_	Excitatory glutamatergic current (AMPA/NMDA) from STN neuron to GPe neuron
*I*^*GABA→STN*^_*ij*_	Inhibitory GABAergic current from GPe neuron to STN neuron
*I*^*AMPAlat*^_*ij*_/*I*^*NMDAlat*^_*ij*_	Excitatory glutamatergic current (AMPA/NMDA) from STN neuron to STN neuron due to collateral synapses
*I*^*GABAlat*^_*ij*_	Inhibitory GABAergic current from GPe neuron to GPe neuron due to collateral synapses
*S*^*GPe*^_*ij*_	Spiking activity of GPe neuron at location (*i*, *j*) at time “*t*”
*S*^*STN*^_*ij*_	Spiking activity of STN neuron at location (*i*, *j*) at time “*t*”
*S*^*Dxy*^_*ij*_	Spiking activity of striatum at location (*i*, *j*) at time “*t*.” The variable “*y*” represents either D1 striatum (=1) or D2 striatum (=2) for an input stimulus “*x*”

TN, Sub Thalamic Nucleus; GPe, Globus Pallidus Externa; GPi, Globus Pallidus Interna.

##### Lateral connections in STN and GPe neurons

Various anatomical studies show the presence of collaterals in STN (Kita et al., 1983) and GPe (Kita and Kita, 1994) neurons. Gillies et al. (2002) show, using a computational model, how various neural firing patterns could emerge due to collaterals in STN (Gillies and Willshaw, 1998). The lateral connections in the current network were modeled as Gaussian neighborhoods. Each neuron (in STN/GPe) receives collateral synaptic input from a fixed number of neighboring neurons located in a 2D grid of size nxn.

#### Effect of DA on synaptic structural plasticity of STN and GPe neurons

Behavioral learning can lead to synaptic structural changes either in dendrites or in signaling pathways (Caroni et al., 2012). Axonal and dendritic spine elongation and reduction in various areas of brain such as neo-cortex and hippocampus is well known (Richards et al., 2005; Gogolla et al., 2007; Caroni et al., 2012). Interestingly, this structural plasticity has also been observed in dorsal and ventral striatum of BG due to DA depletion (Meredith et al., 1995). Structurally, an increase in synaptic strength could be due to increase in the number of contacts or number of dendritic spines (Mckinney, 2010) which is associated with an increase in NMDA current (Tian et al., 2007). The burst firing in STN neurons observed under PD conditions is hypothesized to be due to increased NMDA currents (Zhu et al., 2005; Shen and Johnson, 2010). Also, Robertson et al. (1990) show a reduction in GABA-A receptor expression levels in GPe neurons of MPTP primates (Robertson et al., 1990), an area that receives projections from SNc (Smith and Kieval, 2000). A decrease in GABA-A levels has also been shown to be correlated to decrease in the number of dendritic spines in neurons (Pallotto and Deprez, 2014). A study by Fan et al. (2012) showed a greater proliferation of synapses from GPe to STN neuron in 6-OHDA rats compared to controls (Fan et al., 2012).

Considering the above mentioned experimental results, one may expect dopamine-dependent plasticity in STN and GPe neurons. Experimental studies have shown the synaptic currents from collaterals (inhibitory or excitatory) follow Gaussian distribution (Lukasiewicz and Werblin, 1990). It has been observed during experimental recordings that low DA levels increase the synchrony levels within STN neurons (Bergman et al., 1994, 1998). Theoretically, such behavior can be observed in any excitatory neurons when their lateral connections are strengthened (Hansel et al., 1995). Moreover, GPe neurons also show synchrony (Bergman et al., 1998) at low DA conditions and such a behavior in inhibitory neurons can be observed when their collateral strength is decreased (Wang and Rinzel, 1993). Taking these theoretical and experimental results into account, we assume that the width of the collateral spread to be modulated by DA levels.

Accordingly, the width of Gaussians in STN and GPe laterals (Section Lateral connections in STN and GPe neurons) in the model was assumed to be modulated by DA and modeled as,

(7) $$\begin{array}{l} R_{s}=\frac{r_{s}}{\left(cD21*DA\right)};R_{g}=\frac{r_{g}}{\left(1-cD21*DA\right)};\\ \;w_{ij,pq}^{m\to m}=A_{m}*e^{\frac{-d_{ij,pq}^{2}}{R_{m}^{2}}},\\ \;d_{ij,pq}^{2}={\left(i-p\right)}^{2}+{\left(j-q\right)}^{2} \end{array}$$

(8) $$I_{ij}^{Receplat}=B_{ij}\left(v_{ij}\right)*\sum_{p\;=\;1}^{n}\sum_{q\;=\;1}^{n}w_{ij,pq}^{x\to x}*h_{ij}^{Recep\to x}(t)*(E_{Recep}-V_{ij}^{x}(t))$$

where *r*_*s*_ = constant variance of STN Gaussian, *r*_*g*_ = constant variance of *GPe* Gaussian, *R*_*s*_ = changed variance of STN Gaussian due to the effect of dopamine, *R*_*g*_ = changed variance of *GPe* Gaussian due to the effect of dopamine, cD21 = a constant that determines the effect of DA on STN and *GPe* laterals, *w*^*m*→*m*^_*ij*_ = lateral weight matrix of neuron “*m*” at location (*i,j*), *d*_*ij,pq*_ = distance from center neuron (*p,q*), *R*_*m*_ = *R*_*g*_(or) *R*_*s*_, *A*_*m*_ = strength of lateral synapse, *m* = STN or *GPe* neuron. All parameter values are given in Table 1.

The inhibitory (excitatory) collateral current developed in *GPe* (STN) neurons are governed by Equation (8). The effect of “*B*_*ij*_” is valid only for NMDA synapses made by STN collaterals but not for GABAergic synapses.

Experimental data suggests that DA causes post-synaptic effects on glutamatergic and GABA currents in STN and GPe respectively (Smith and Kieval, 2000; Cragg et al., 2004; Fan et al., 2012). Thus, we included a factor (cd2), which regulates the effect of the DA, on synaptic currents between STN and GPe, as in Equation (9). This leads to a decrease in the regulated current with increase in DA, as observed in Kreiss et al. (1997).

(9) $$W_{x\to y}=\left(1-cd2*DA\right)*w_{x\to y}$$

where the synapses are *GPe*→STN and STN→*GPe*. A similar method for DA-dependent synaptic modulation on striatal neurons was used in Humphries et al. (2009).

#### Total synaptic currents received by each neuron

##### Total synaptic currents received by GPe neurons.

The total synaptic current received by a *GPe* neuron at lattice position (*i*, *j*) is the summation of GABAergic input from the D2-expressing striatal MSNs (Gerfen et al., 1990) Equation (5), glutamatergic current from STN considering both AMPA and NMDA currents (Götz et al., 1997) Equation (5) and the inhibitory lateral current form other *GPe* neurons Equation (8). The influence of DA on the GABAergic current from D2 striatum to *GPe* neuron (Hadipour-Niktarash et al., 2012) was accounted by the variable cD2.

(10) $$I_{ij}^{GPesyn}=I_{ij}^{GABAlat}+I_{ij}^{NMDA\to GPe}+I_{ij}^{AMPA\to GPe}+\;I_{ij}^{StrD2\to GPe}*cD2$$

(11) $$cD2=\frac{A_{D2}}{1+\exp(\lambda^{Str}*DA)}$$

where, *I*^*GABAlat*^_*ij*_ = the inhibitory lateral GABAergic current from other *GPe* neurons, *I*^*NMDA→GPe*^_*ij*_ = excitatory glutamatergic current from STN neuron due to NMDA receptor, *I*^*AMPA→GPe*^_*ij*_ = excitatory glutamatergic current from STN neuron due to AMPA receptor, *I*^*StrD*2→*GPe*^_*ij*_ = inhibitory GABAergic current from D2 striatum, cD2 = Gain parameter that affects the GABAergic D2 striatal current.

##### Total synaptic currents received by STN neurons.

The total synaptic current received by an STN neuron at lattice position (*i*, *j*) is summation of GABAergic current from GPe neurons (Fan et al., 2012) Equation (5) and glutamatergic input (both AMPA and NMDA) from other STN (Kita et al., 1983) Equation (8).

(12) $$I_{ij}^{STNsyn}=I_{ij}^{GABA\to STN}+I_{ij}^{NMDAlat}+I_{ij}^{AMPAlat}$$

Where, *I*^*NMDAlat*^_*ij*_ = excitatory glutamatergic current from collateral STN neurons due to NMDA receptor, *I*^*AMPAlat*^_*ij*_ = excitatory glutamatergic current from collateral STN neurons due to AMPA receptor, *I*^*GABA→STN*^_*ij*_ = inhibitory GABAergic current from *GPe* neuron.

##### Total synaptic currents received by GPi neurons.

The total synaptic current received by a GPi neuron at lattice position (*i,j*) is a summation of GABAergic currents from D1 striatal neurons (Gerfen et al., 1990)and glutamatergic (both AMPA and NMDA) input from STN neurons (Gerfen and Surmeier, 2011). The increase in GABAergic current from D1 striatum to GPi neurons due to DA modulation (Kliem et al., 2007) was taken into account by the variable *cD*1.

(13) $$I_{ij}^{GPisyn}=I_{ij}^{NMDA\to GPi}+I_{ij}^{AMPA\to GPi}+I_{ij}^{StrD1\to GPi}*cD1$$

(14) $$cD1=\frac{A_{D1}}{1+\exp(-\lambda^{Str}*(DA-1))}$$

Where, *I*^*NMDA→GPi*^_*ij*_ = excitatory glutamatergic current from STN neuron due to NMDA receptor, *I*^*AMPA→GPi*^_*ij*_ = excitatory glutamatergic current from STN neuron due to AMPA receptor, *I*^*StrD*1→*GPi*^_*ij*_ = inhibitory GABAergic current from D1 striatum, *cD*1 = Gain parameter that affects the GABAergic striatal current.

### Synchronization

The phenomenon of neural synchrony has attracted the attention of many computational and experimental neuroscientists in the recent decades (Pinsky and Rinzel, 1995; Plenz and Kital, 1999; Hauptmann and Tass, 2007; Kumar et al., 2011; Park et al., 2011). It is believed that partial synchrony helps in the generation of various EEG rhythms such as alpha and beta (Izhikevich, 2007). Studying synchrony in neural networks has been gaining importance due to its presence in normal functioning (coordinated movement of the limbs) and in pathological states (e.g., synchronized activity of CA3 neurons in the hippocampus during an epileptic seizure) (Pinsky and Rinzel, 1995). Plenz and Kitai (1998) proposed that STN-GPe might act as a pacemaker (Plenz and Kital, 1999), a source for generating oscillations in pathological conditions such as Parkinson's disease. Park et al. (2011) report the presence of intermittent synchrony between STN neurons and its Local field potentials (LFP), recorded using multiunit activity electrodes from PD patients undergoing DBS surgery (Park et al., 2011). They also calculated the duration of synchronized and desynchronized events in neuronal activity by estimating transition rates, which were obtained with the help of first return maps plotted using phase of neurons (Park et al., 2010, 2011). To observe how dopamine changes synchrony in STN-GPe, we calculated the phases of individual neurons as defined in (Pinsky and Rinzel, 1995).

The phase of *j*th neuron was calculated as follows,

(15) $$\emptyset_{j}\left(t\right)=2*\pi *\frac{\left(T_{j,k}-t_{j,k}\right)}{\left(t_{j,k+1}-t_{j,k}\right)}$$

(16) $$R^{sync}\left(t\right)*e^{i\theta \left(t\right)}=\frac{1}{N}\sum_{j\;=\;1}^{N}{\text{e}}^{\text{i}\emptyset_{j}\left(t\right)}$$

where, *t*_*j,k*_ and *t*_*j,k*+1_ are the onset times of *k*th and *k* + 1th spike of the *j*th neuron *T*_*j,k*_ ∈ [*t*_*j,k*_, *t*_*j,k*+1_], ∅_*j*_ (*t*) = Phase of *j*th neuron at time “*t*,” R^sync^ is the synchronization measure 0 ≤ R^sync^ = 1,θ = Average phase of neurons, *N* = total number of neurons in the network.

### Action selection using the race model

Action selection is modulated by BG output nucleus GPi which projects back to the cortex via the thalamus. We have used the race model (Vickers, 1970) for the final action selection where an action is selected when temporally integrated neuronal activity of the output neurons crosses a threshold (Frank, 2006; Frank et al., 2007; Humphries et al., 2012).

The dynamics of the thalamic neurons is as follows,

(17) $$\frac{dz_{k}\left(t\right)}{dt}=-z_{k}\left(t\right)+f_{Gpik}(t)$$

(18) $$\begin{array}{l} {f^{\prime }}_{Gpik}=\frac{1}{(N*N)/k}\sum_{t=1}^{T}\left(\sum_{i\;=\;1}^{N}\sum_{j=1}^{N/k}S_{ij}^{GPik}(t)\right)\\ f_{GPik}=\frac{f_{GPi}^{\max}-{f^{\prime }}_{Gpik}}{f_{GPi}^{\max}} \end{array}$$

where, *z*_*k*_ (*t*) = integrating variable for *k*th stimulus, *f*_GPik_ (*t*) = normalized and reversed average firing frequency of *GPi* neurons receiving *k*th stimulus from striatum, *f*^maxGPi^ = highest firing rate among the *GPi* neurons, *S*^*Gpik*^_*ij*_ = neuronal spikes of *GPi* neurons receiving *k*th stimulus, *N* = number of neurons in a single row/column of *GPi* array (=50), *T* = duration of simulation.

The first neuron (*z*_*k*_) among *k* stimuli to cross the threshold (=0.15) represents the action selected. All the variables representing neuron activity are reset immediately after each action selection.

### Binary action selection task

The first task we simulated was the simple binary action selection similar to Humphries et al. (2006), where two competing stimuli were presented to the model (Humphries et al., 2006). The input firing frequency is thought to represent “saliency,” with higher frequencies representing higher salience (Humphries et al., 2006). The response of striatal output to cortical input falls in the range of a few tens of Hz (Sharott et al., 2012). Therefore the frequencies that represent the 2 actions were assumed to be around 4 Hz (stimulus #1) and 8 Hz (stimulus #2). Spontaneous output firing rate of the striatal neurons (without input) is assumed to be around 1 Hz (Plenz and Kitai, 1998; Sharott et al., 2012). Selection of higher salient stimulus among the available choices could be considered as “exploitation” while selecting the less salient one as “exploration” (Sutton and Barto, 1998). So the action selected is defined as “Go” if stimulus #2 (more salient) is selected, “explore” if stimulus #1(less salient) is selected and “No Go” if none of them is selected.

**Figure 2:**
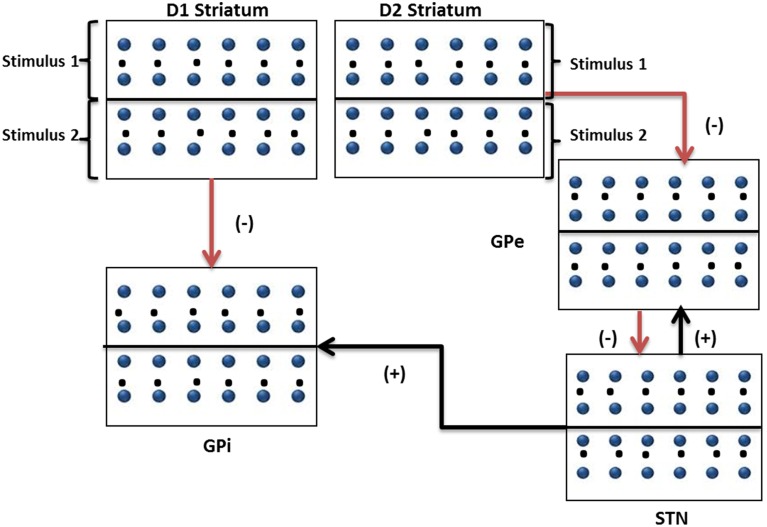
**Modeling the binary action selection task.** The figure shows a pictorial representation of inputs to striatal, GPi, GPe, and STN networks (50^*^50) depicting D1 and D2 neuronal pools and their projections to GPi and GPe networks. Stimulus #1 and Stimulus #2 are the inputs with frequencies representing saliency.

The inputs were given spatially such that the neurons in the upper half of the lattice receive stimulus #1 and lower half the other (Figure 2). The striatal outputs from D1 and D2 neurons of the striatum are given as input to GPi and GPe modules respectively with the projection pattern as shown in Figure 2. Poisson spike trains corresponding to Stimulus #1 were presented as input to neurons (1–1250) and were fully correlated among themselves. Similarly, Poisson spike trains corresponding to Stimulus #2 were presented as input to neurons (1251–2500) and were fully correlated among themselves. Stimulus #1 and #2 are presented for an interval of 100 ms between 100 and 200 ms; at other times uncorrelated spike trains at 1 Hz are presented to all the striatal neurons. The values of the parameters used synaptic weight to implement the binary action selection problem are given in Table 1.

### The N-armed bandit task

We now describe the 4-arm bandit task (Daw et al., 2006; Bourdaud et al., 2008) used to study exploratory and exploitatory behavior. In this experimental task, subjects were presented with 4 arms where one among them is to be selected in every trial for a total of 300 trials. The reward/payoff for each of these slots was obtained from a Gaussian distribution whose mean changes from trial to trial with payoff ranging from 0 to 100. The payoff, *r*_*i.k*_ associated with the *i*th machine at the *k*th trial was drawn from a Gaussian distribution of mean μ_*i,k*_ and standard deviation (SD) σ_0_. The payoff was rounded to the nearest integer, in the range [0, 100]. At each trial, the mean is diffused according to a decaying Gaussian random walk. The trial was defined as an “exploitatory” trial if highest reward giving arm was selected else defined as an “exploratory” trial.

The payoffs generated by the slot machines are computed as follows,

(19) $$\mu_{i,k+1}=\lambda_{m}\mu_{i,k}+(1-\lambda_{m})\theta_{m}+e$$

(20) $${r^{\prime }}_{i,k}\approx N(\mu_{i,k},\sigma_{0}^{2})$$

(21) $$r_{i,k}=round({r^{\prime }}_{i,k})$$

where, μ_*i,k*_ is the mean of the Gaussian distribution with standard deviation(σ_0_) for *i*th machine during *k*th trial. λ_*m*_ and θ_*m*_ control the random walk of mean (μ_*i,k*_) and *e* ~ *N*(0, σ^2^_*d*_) is obtained from Gaussian distribution of mean 0 and standard deviation σ_*d*_. *r*_*i,k*_ and *r*'_*i,k*_ are the payoffs before and after rounding to nearest integer respectively. The initial value of mean payoff, μ_*i*,0_, is set to a value of 50. All the values for the parameters λ_*m*_, θ_*m*_, σ_*d*_, σ_0_ were adapted from (Bourdaud et al., 2008).

To make an optimal decision, the subjects need to keep track of rewards associated with each of the 4 arms. The subject's decision to either explore or exploit would depend on this internal representation which would closely resemble the actual payoff that is being obtained. It is quite difficult to identify whether the subject made an exploratory decision or an exploitative one just by observing the EEG and selected slot data. A subject-specific model is required to classify their decisions and identify the strategy (Daw et al., 2006; Bourdaud et al., 2008). Keeping this in mind, Bourdaud et al. (2008) used a “behavioral model” that uses the soft-max principle of RL to fit the selection pattern of human subjects. The parameter “β” of the behavioral model was adjusted such that the final selection pattern matches that of individual subjects in the experiment (refer Appendix A and Table A.1 in Supplementary Material for details). The parameter “β” which controls the exploration level in the behavioral model is tuned to match % exploitation obtained for each of the 8 subjects (1 subject's data was discarded because of artifacts); 2 out of the 8 subjects had similar exploration levels. Hence, a total of 6 subjects' data is taken to account to check the performance of the proposed spiking BG model.

#### Strategy for slot machine selection

To simulate the experiment, we utilized the concepts of RL and combined the dynamics of BG model to select an optimally rewarding slot in each trial. Experimental data show that BG receives reward related information in the form of dopaminergic input to striatum (Niv, 2009; Chakravarthy et al., 2010). Cortico-striatal plasticity changes due to dopamine (Reynolds and Wickens, 2002) were incorporated in the model by allowing DA signals modulate the Hebb-like plasticity of cortico-striatal synapses(Surmeier et al., 2007).

**Figure 3:**
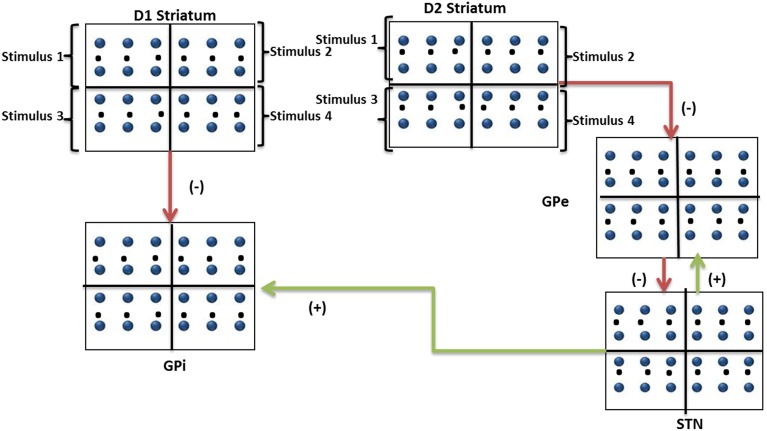
**Modeling the n-armed bandit task**. The figure shows a pictorial representation of inputs to striatal, GPi, GPe, and STN networks (50^*^50) depicting D1 and D2 neuronal pools and their projections to GPi and GPe networks. Stimulus #1, Stimulus #2, Stimulus #3, and Stimulus #4 are the 4 arms whose saliencies are represented in their cortico-striatal weights.

The architecture of the proposed network model is depicted in Figure 3. The output of striatum (both D1 and D2 parts) was divided equally into 4 quadrants which receive input from corresponding stimulus. The stimuli are associated with 2 weights (*w*^*D*1^_*i*,0_, *w*^*D*2^_*i*,0_) initialized with equal value of 50 which represent the cortico-striatal weights of D1 and D2 MSNs in the striatum. Each of the cortico-striatal weight represents the saliency (in terms of striatal spike rate) for that corresponding arm. These output spikes generated from each of the D1 and D2 striatum project to GPi and GPe respectively. The final selection of an arm is made as in Section Action Selection Using the Race Model. The reward *r*_*i,k*_ received for the selected slot was sampled from Gaussian distribution with mean μ_*i,k*_ and SD (σ_0_) Equation (19).

Using the obtained reward (*r*_*i,k*_), the expected value of the slots, inputs to D1 and D2 striatum are updated using the following equations:

(22) $$\Delta w_{i,k+1}^{D1}=\eta \delta_{k}x_{i,k}^{inp}$$

(23) $$\Delta w_{i,k+1}^{D2}=-\eta \delta_{k}x_{i,k}^{inp}$$

The expected value (V_*k*_) for *k*th trial is calculated as

(24) $${\text{V}}_{\text{k}}=\sum_{i=1}^{4}w_{i,k}^{D1}*x_{i,k}^{inp}$$

The received payoff (Re_*k*_) for *k*th trial is calculated as

(25) $${\text{Re}}_{\text{k}}=\sum_{i=1}^{4}r_{i,k}*x_{i,k}^{inp}$$

The error (δ) for *k*th trial is defined as

(26) $$\delta_{\text{k}}={\text{Re}}_{\text{k}}-{\text{V}}_{\text{k}}$$

where, *w*^*D*1^_*i,k*_ are the cortico-striatal weights of D1 striatum for *i*th machine in *k*th trial, *w*^*D*2^_*i,k*_ are the cortico-striatal weights of D2 striatum for *i*th machine for *k*thtrial, *r*_*i,k*_ is the reward obtained for the selected *i*th machine for *k*th trial, *x*^*inp*^_*i,k*_ is the binary input vector representing the 4 slot machines, e.g., if the first slot machine is selected *x*^*inp*^_*i,k*_ = [1 0 0 0], η (=0.3) is the learning rate of D1 and D2 striatal MSNs, Re_*k*_ is the recieved payoff for selected slot for *k*th trial,V_*k*_ is the expected value for selected slot for *k*th trial

The cortico-straital weights are updated Equations (22) and (23) using the error term “δ” Equation (26). The reward related information in the form of dopaminergic input to striatum has been correlated to the error (δ) (Niv, 2009; Chakravarthy et al., 2010). The δ calculated from the Equation (26) has both positive and negative values with no upper and lower boundaries but the working DA range in the model was limited to small positive values (0.1–0.9). Hence, a mapping from δ to DA is defined as follows,

(27) $$DA=sig(\lambda *\delta_{k})$$

where, *DA* is the dopamine signal within range of 0.1–0.9, λ is the slope of sigmoid (=0.2), δ_*k*_ is the error obtained for *k*th trial Equation (26), sig () is the sigmoid function given by equation:

(28) $$\text{sig}(\text{y})=\frac{1}{1+e^{-y}}$$

To verify whether a rewarding slot is selected, delta (δ_*k*_) as described in Equation (26) was calculated which keeps track of expected and actual payoff.

## Results

**Figure 4:**
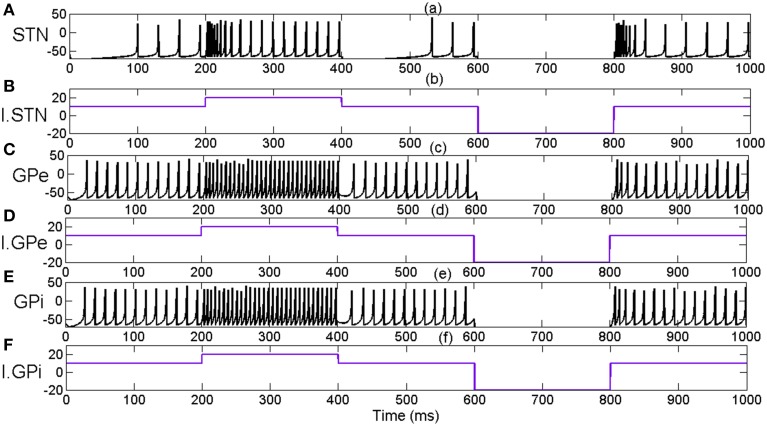
**Indicates the effect of external applied current (*****I***^***x***^_***ij***_**) on neuronal firing pattern and rates of STN, GPe and GPi neurons. (A)** Membrane potential of STN neuron for applied current **(B)**, I.STN. **(C)** Membrane potential of GPe neuron for applied current **(D)**, I.GPe **(E)** Membrane potential of GPi neuron for applied current **(F)**, I.GPi. X-axis indicates the time of simulation in milliseconds.

We have investigated if the chosen Izhikevich parameters for STN, GPe and GPi displayed biological properties of corresponding neurons (Figure 4). The distinctive property of inhibitory rebound in STN (Hamani et al., 2004) was observed in simulation which was absent in GPe and GPi neurons. The firing rate of STN, GPe and GPi neurons increased when increasing current inputs (*I*^*x*^_*ij*_) as observed in experimental recordings (Magnin et al., 2000; Thibeault and Srinivasa, 2013).

We then present results from 3 sets of simulation studies starting with the characterization of the dynamics of STN-GPe network (Simulation set 1). A key idea explored in this study is that the dynamics of STN-GPe critically influence action selection by BG, particularly in the component of exploration. Therefore, we characterize STN-GPe network dynamics in terms of firing frequency and synchronization measure, R^sync^. Following that, we present results from the simple binary action selection task (Simulation set 2) where the presence of 3 regimes (Go/explore/No-Go) in action selection is demonstrated revealing the interplay of IP and DP in action selection. Then we present results from the n-arm bandit task (Simulation set 3). The amount of exploration obtained from experimental data was comparable to that of BG model by changing the IP weight.

### Simulation set 1: STN-GPe circuit dynamics and synchrony

Pathological oscillations of STN and GP have been associated with various PD symptoms (Bergman et al., 1994; Brown et al., 2001; Levy et al., 2002; Brown, 2003; Litvak et al., 2011; Park et al., 2011; Dovzhenok and Rubchinsky, 2012). Correlated neural firing patterns in STN and GPi can be seen in both experimental conditions of dopamine depletion and in Parkinsonian conditions (Bergman et al., 1994; Nini et al., 1995; Magnin et al., 2000; Brown et al., 2001). Using a conductance based model of STN and GPe system, Terman et al. (2002) demonstrated a variety of rhythmic behaviors by varying the connectivity patterns between STN and GPe (Terman et al., 2002). In the present model we assume that the connections within and between STN and GPe are dopamine-dependent (Cragg et al., 2004) and show increased synchronized behavior under conditions of reduced dopamine, resembling the situation in dopamine-deficient conditions of Parkinson's disease. No inputs were given to STN-GPe network; dopamine (DA) was varied as a parameter Equations (7) and (9) and neural dynamics in the two systems was studied.

**Figure 5:**
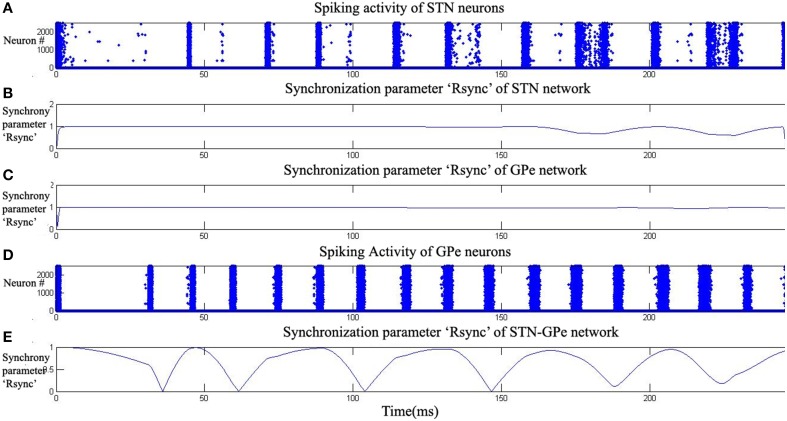
**Highly synchronized activity of STN-GPe system at low dopamine level (DA = 0.1).** Plots **(A,D)** raster plots indicate the activity of STN and GPe neurons with time. Plots **(B,C,E)** indicate the synchronization parameter (*R*^*sync*^) calculated for STN, GPe and STN-GPe respectively.

**Figure 6:**
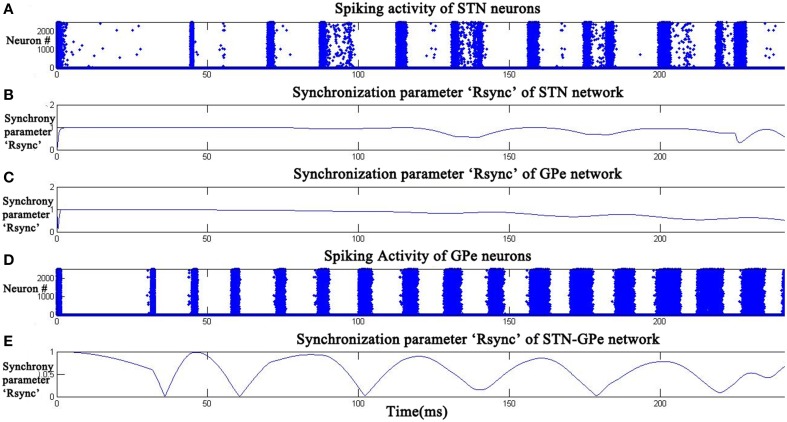
**STN-GPe Network became desynchronized at intermediate dopamine level (DA = 0.5).** Raster plots **(A,D)** indicate the activity of STN and GPe neurons with time. Plots **(B,C,E)** indicate the synchronization parameter (*R*^*sync*^) calculated for STN, GPe, and STN-GPe respectively.

**Figure 7:**
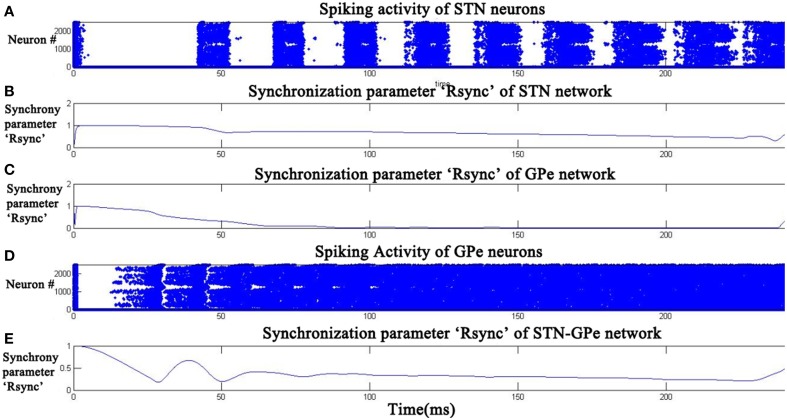
**Desynchronized activity of STN- GPe neurons at high dopamine level (DA = 0.9).** Raster plots **(A,D)** indicate the activity of STN and GPe neurons with time. Plots **(B,C,E)** indicate the synchronization parameter (R^*sync*^) calculated for STN, GPe, and STN-GPe respectively.

The firing patterns in both STN and GPe varied from synchronized to desynchronized states as levels of dopamine are increased from 0.1 (low) to 0.9 (high) (Figures 5–**7**). Synchronization parameter “*R*^sync^” as described in Section Action Selection Using the Race Model, Equation (16), is calculated within STN (*R*^*sync*^_*STN*_) neurons, GPe (*R*^*sync*^_*GPe*_) neurons and also between STN and GPe (*R*^*sync*^_*STNGPe*_) neurons (**Figure 9**). For low value of DA (0.1), we observed that both *R*^*sync*^_*STN*_ (Figure 5B) and *R*^*sync*^_*GPe*_ (Figure 5C) were high (=1). The value of *R*^*sync*^_*STNGPe*_ (Figure 5E) oscillated between 0 and1 indicating an alternating pattern of synchrony, which is observable in raster plots (Figures 5A,D).

As DA value was increased to an intermediate level (0.5), a decrease in the value of *R*^*sync*^_*STN*_ (Figure 6B) and *R*^*sync*^_*GPe*_ (Figure 6C) was observed with time. The decrement in the amplitude of oscillatory pattern in *R*^*sync*^_*STNGPe*_ (Figure 6E) indicates the presence of synchronized and desynchronized firing patterns of the neurons. This can be observed in the raster plots of STN and GPe neurons (Figures 6A,D) which show the beginning of desynchronized behavior.

At high DA (0.9), *R*^*sync*^_*STN*_ (Figure 7B) has decreased to an average value of 0.3 and *R*^*sync*^_*GPe*_ (Figure 7C) reached an average value of 0.1. The oscillatory pattern in *R*^*sync*^_*STNGPe*_ (Figure 7E) is completely absent at high DA indicating a desynchronized firing pattern, which can also be seen in the raster plots of STN and GPe neurons (Figures 7A,D).

The average firing rate of the neurons in the network was calculated using the following equation:

(29) $$f_{k}=\frac{1}{\left(N*N\right)}\sum_{t=1}^{T}\left(\sum_{i\;=\;1}^{N}\sum_{j\;=\;1}^{N}S_{ij}^{k}(t)\right)$$

where, *k* = denotes STN/GPe, *f* = average firing rate of the STN/GPe network for a simulation time of 1 s, *S*^*k*^_*ij*_(*t*) = Spiking activity of neuron at (*i,j*) in the network defined by “*k*,” *N* = total number of neurons (50∗50), *T* = simulation time (1 s).

**Figure 8:**
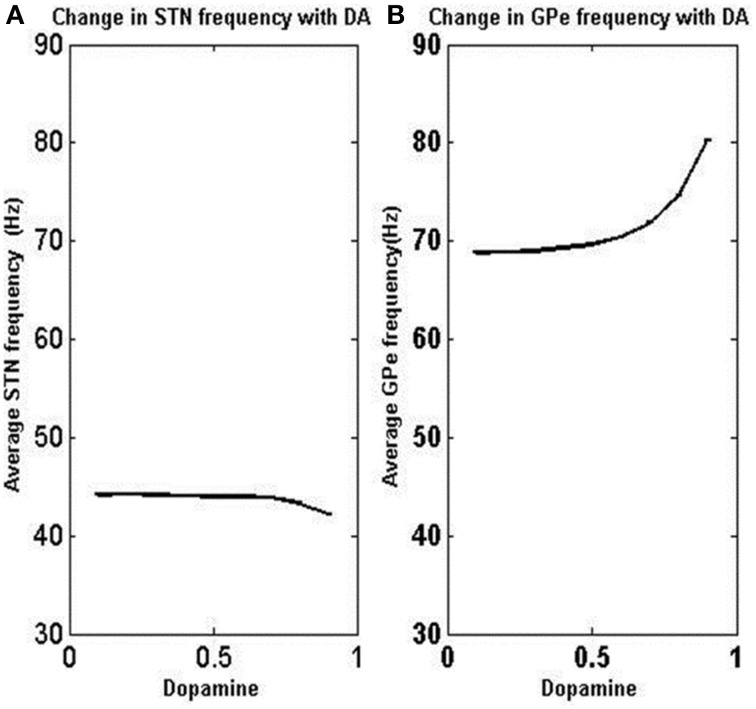
**Variation of average firing rate of STN and GPe neurons with DA levels.** As the dopamine level is increased, the firing rate increased in GPe neurons but decreased in STN neurons. X-axis indicates the level of dopamine and y-axis is the firing rate of respective neurons. **(A)** Shows the change in STN frequency with increase in DA level. **(B)** Shows the change in GPe frequency with increase in DA level.

The firing rate of STN neurons decreased from a range of 45–50 Hz (due to bursting) at low DA(0.1) to 35–40 Hz at high DA (0.9) (Figure 8A). Similarly the frequency of GPe neurons increased from about 60–70 Hz at low DA (0.1) to 80–90 Hz at high DA (0.9) (Figure 8B). The simulated firing rates of STN and GPe neurons match with reports from electrophysiological studies (Magnin et al., 2000; Benazzouz et al., 2002) where an increase and decrease in firing rate was observed for STN and GPe respectively in Parkinsonian conditions and vice versa for normal conditions.

**Figure 9:**
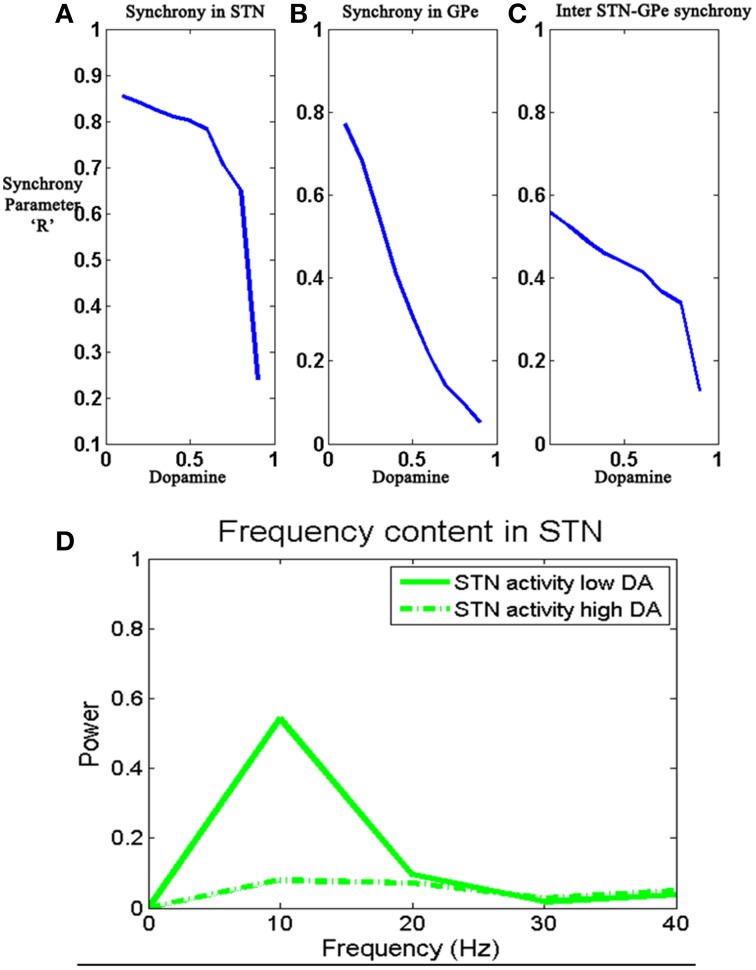
**Shows the change in the 3 synchronization values** ***R***^***sync***^_***STN***_ **(A),** ***R***^***sync***^_***GPe***_ **(B), and** ***R***^***sync***^_***STNGPe***_ **(C) oscillatory activity in STN neurons (D) with the value of DA (0.1–0.9).** Simulations show reduced synchronization within STN and GPe networks, and also between STN and GPe networks, as DA is increased.

Under low DA conditions, the contribution of excitatory lateral current is higher in STN, thereby increasing overall firing rate (Figure 8A) and synchrony (Figure 9A) which is observed in general excitatory neurons (Hansel et al., 1995) and such synchrony was found to be absent at high DA levels. GPe neurons show a synchronized firing pattern with decreased lateral synaptic weights at low DA level (Figure 9B) (Wang and Rinzel, 1993). On the contrary, a high lateral inhibition at high DA prevents the neighborhood neurons from firing at the same time, thereby producing a desynchronized firing pattern.

The effect of DA on the synchronization of STN and GPe neurons was studied by estimating the values of*R*^*sync*^_*STN*_, *R*^*sync*^_*GPe*_ *R*^*sync*^_*STNGPe*_ for increasing values of DA (0.1–0.9). The 3 “*R*^*sync*^” values showed a decrease in amplitude with an increase in DA level (Figures 9A–C) and the oscillatory activity at low and high DA levels was shown in Figure 9D. Under low DA conditions, GPe activity follows STN activity (Plenz and Kital, 1999) thus forming a pacemaker kind of circuit, which could be the source of STN-GPe oscillations. One of the suspected reasons of bursting activity in STN is the decreased inhibition from GPe neurons (Plenz and Kital, 1999) at low DA levels. This feature is captured by the model since GPe firing rates are smaller for lower DA levels. The STN neurons showed oscillatory around the frequency of 10 Hz at low DA but was absent at high DA level (Kang and Lowery, 2013).

### Simulation set 2: binary action selection

The simulation was run for a period of 250 ms, out of which the input stimuli (assumed to be projected from cortex) is given during the time interval 100–200 ms. A background input around 1 Hz was given during the rest of the simulation. The striatal network with 2500 neurons is divided into two equal sections such that the neurons in the first section (1–1250) received Stimulus #1 and the rest(1251–2500) received Stimulus #2 (Figure 2). Seeking to understand how dopamine affects action selection, we varied the dopamine level from 0.1 (low) to 0.9 (high) and observed which of the 2 inputs was selected at different dopamine levels. The action selected is classified into Go/Explore/No-Go depending on which stimulus is selected: it is “Go” if the stimulus with higher salience is selected, “Explore” if the other stimulus is selected, and “No-Go” if no input is selected.

**Figure 10:**
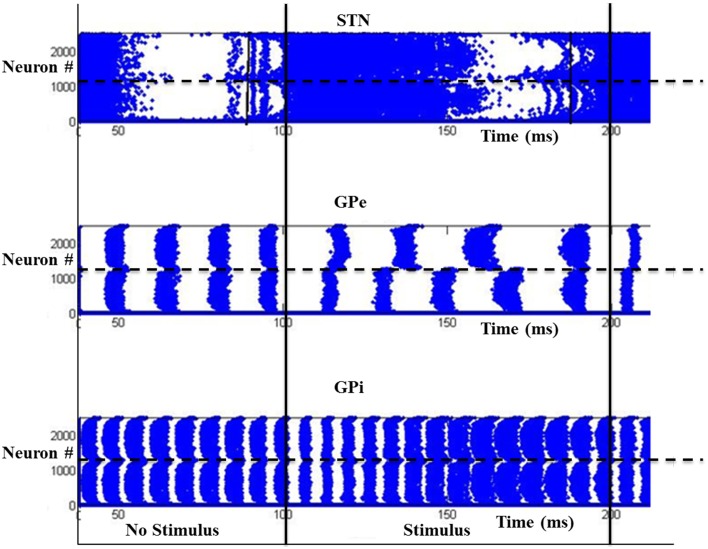
**Activity of the model at low DA (=0.1) with high firing rate in GPi activity where x-axis represent the time of spiking and y-axis is the spatial representation of 2500 neurons.** S1 and S2 are the two stimuli given spatially to the striatum.

We studied the pattern of action selection as a function of DA level. In low DA state, the activity of STN is high (Figure 10) thus increasing the activity of GPi (Figure 10); an overactive GPi inhibits the thalamus and therefore no action is selected (Figure 10). We thus have a “No-Go” case under low DA conditions.

**Figure 11:**
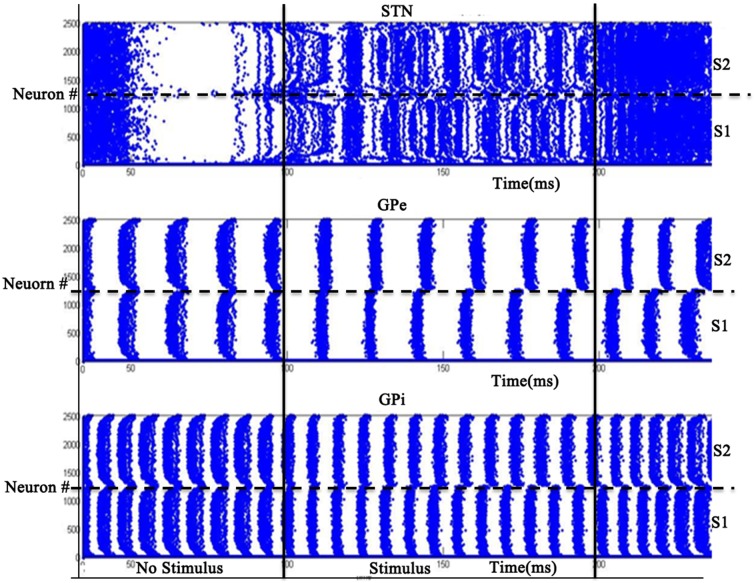
**Model Activity at intermediate DA (=0.5) with alternating behavior in GPi activity.** Where x-axis represents the time of spiking and y-axis is the spatial representation of 2500 neurons. S1 and S2 represent the 2 stimuli given to the network spatially.

At intermediate levels of dopamine (0.4–0.6), GPi neurons dynamically segregate into two pools, those corresponding to Stimulus #1 and #2 respectively. Neurons in either pool fire in strong synchrony among themselves, while the two pools fire in alternation (Figure 11). The alternation is more visible in GPe and GPi and not so much in STN. This alternation, as we will see below, introduces variability in action selection, even though there is no change in input stimulus. We interpret this variability as a form of exploration in action selection because the burst of activity for the neuron pool corresponding to one action increases the probability of its selection, while simultaneously preventing the selection of the other action. We interpret this dynamical regime corresponding to intermediate DA levels as the “explore” regime.

**Figure 12:**
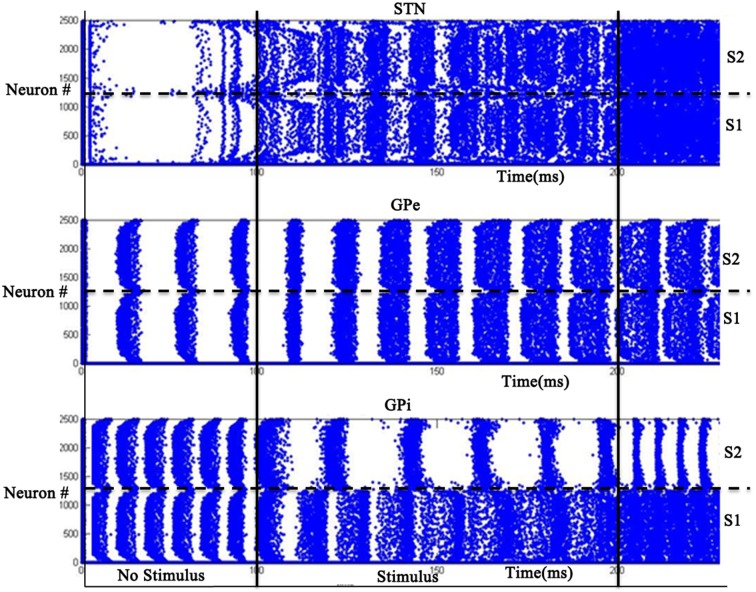
**Model Activity at high DA (=0.9) state where x-axis represents the time of spiking and y-axis is the spatial representation of 2500 neurons.** S1 and S2 represent the 2 stimuli which were given spatially to the network.

For high dopamine levels (Figure 12), the activity of D1 striatum is high and the DP dominates the IP. The stronger input (Stimulus #2) is selected always as it reaches the threshold sooner. Thus higher dopamine levels correspond to the “Go” regime.

**Figure 13:**
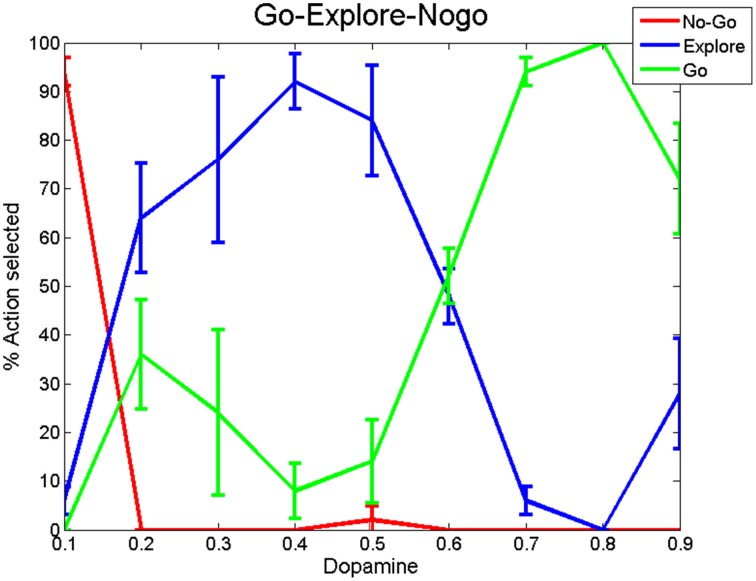
**Percentage of action selection observed in the Go, No-Go, and Explore regimes averaged over 200 trials with DP and IP weight values at w_STN→GPi_ = 1.15 and w_Str→GPi_ = 0.8.** We ran the simulation for 100 trials and segmented in to 4 bins (25 trials each). We then calculated the variance of each regime across all DA levels.

Simulations were run for 100 trials and the percentage of actions selected under each regime (Go, Explore and No-Go) was calculated for dopamine levels ranging from low (0.1) to high (0.9) (Figure 13). From Figure 13, we may note that the probability of No-Go, where no action is selected, decreases with increase in dopamine; probability of Go increases with dopamine; the peak of exploration is found at intermediate levels of dopamine.

To check the influence of other structures on action selection, we removed the STN projections to GPi, which is best done by omitting the first two terms on the right side of Equation (13). The resulting decision plot exhibited only Go and No-Go with a completely flat Explore regime (Figure B1-b in Supplementary Material Appendix B). The above result suggests that the IP is crucial for exploration. Then we studied how various aspects of the STN-GPe network affect exploration. Changes in GPe lateral connections did not alter exploration levels (results not shown). We then varied the strength of STN-lateral connections and found that at very high values, the system shows very little exploration (Figure B1-a in Supplementary Material Appendix B). STN lesions in 6-OHDA and MPTP animals are known to relieve the symptoms of PD and initiate motor movements (Baunez et al., 1995) but results in multiple deficits while performing attentional and choice tasks such as increase in reaction time and decrease in exploration levels (Baunez et al., 1995, 2001; Baunez and Robbins, 1997). So we studied the effect of STN lesions on exploration and found that as the size of lesion is increased the amount of exploration decreased. We have added the result for a lesion patch of 20 × 20 neurons (Figure B1-c in Supplementary Material) where the lesion was created at the center of the STN neuron lattice. This was achieved by setting the spiking activity of the corresponding neurons to zero (=0). To investigate the effect of STN laterals on exploration, we increased the strength of STN laterals and calculated % exploration at intermediate levels [0.4–0.6] by applying the binary action selection problem. We increased strength of STN laterals from [0.05 to 0.25] with a step of 0.05. We have observed that at low and high levels, the system does not show exploration but peaks for a range of strengths. The result is shown in Figure B2 in Supplementary Material Appendix B.

### Simulation set 3: the N-armed bandit task

The decision making ability of the BG model was checked by comparing its performance with behavioral model, representation of experimental data in the n-armed bandit task (*n* = 4). The task was simulated for a total of 300 trials. The payoff pattern of the 4 arms for 300 trials calculated using the Equations (19)–(21) is shown in Supplementary Material Figure A1.

#### Parameter “delta”

The difference between the received payoff and estimated payoff from the BG model, the error (δ^*bg*^_*k*_) was calculated for each trial. These results were compared with the error (δ^*be*^_*k*_) obtained from the behavioral model (Bourdaud et al., 2008). The performance of BG model was found to be comparable to behavioral model, which was reflected in the difference between the expected values (V) obtained from behavioral model and the BG model, defined as *e*^*bebg*^_*k*_ = *V*^*bg*^-*V*^*be*^, where *V*^*bg*^ and *V*^*be*^ are expected values obtained from BG and behavioral model. The average and SD of the 3 errors (δ^*bg*^_*k*_, δ^*be*^_*k*_, *e*^*bebg*^_*k*_) obtained by simulating both behavioral and BG model are listed in Table 3 for all the 6 subjects.

**Table 3:** **Errors obtained from behavioral model (δ**^***be***^_***k***_) **and BG (δ**^***bg***^_***k***_) **model independently and a comparison of the errors obtained from the 2 models (*****e***^***bebg***^_***k***_**)**.

**Subject**	**δ^*bg*^**	**δ^*be*^**	***e*^*bebg*^**
1	4.68 ± 4.74	5.27 ± 4.36	4.40 ± 4.49
2	5.04 ± 4.15	5.80 ± 4.59	3.95 ± 4.02
3	5.52 ± 5.05	5.88 ± 6.10	4.27 ± 6.12
4	5.05 ± 4.15	5.18 ± 4.27	4.31 ± 4.20
5	4.89 ± 4.13	5.85 ± 5.21	4.63 ± 4.51
6	4.45 ± 3.52	6.06 ± 5.55	4.68 ± 4.55

*Column 2 shows error (δ^bg^_k_) values for each of the 6 subjects in BG model. Column 3 shows the error obtained from behavioral model (δ^be^_k_) of Bourdaud et al. (2008). Column 4 indicates the difference in error (e^bebg^_k_) which is the difference between the expected values (V^bg^, V^be^) calculated from the 2 models*.

δ bg, error obtained from BG model; δ be, error obtained from Behavioral model; ebebg, difference between Vbg and Vbe.

#### Percent exploitation

In addition to payoff error (δ), another measure that we used to compare performance of BG model with the experimental data, which measures “percentage exploitation.” It is defined as the percentage number of times the highest (expected) reward yielding action (calculated over 300 trials) was selected. For example in a trial “*k*” if highest reward is obtained from slot 4, and if the model also selects slot 4 then the trial resulted in exploitation; else it is exploration. We calculated the average percentage exploitation values for 10 sessions, where each session consists of 300 trials.

Subject to subject exploration variability was accounted by varying the “temperature” parameter β in the behavioral model Equation (A.8) (Appendix A in Supplementary Material). The parameter “β” controls the exploit-explore balance (higher β implies greater exploitation). Since the indirect pathway (IP) dynamics drives exploration in the BG model, we expected that varying the strengths of the direct pathway (DP) (decreasing w_Str→GPi_) and the indirect pathway (increasing w_STN→GPi_) would give similar results in terms of decreased % exploitation levels.

**Figure 14:**
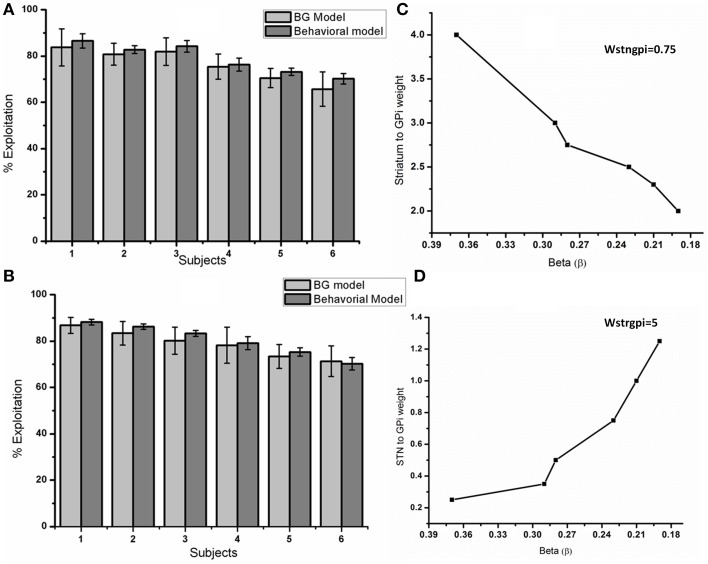
**Compares the performance of BG model with the behavioral model. (A)** Shows the percentage exploitation obtained for each of the 6 subjects from BG and behavioral model. Y axis represents percentage exploitation and X axis represents a subject which is a specific beta value (β) in behavioral model and the DP weight (w_Str→ GPi_) in the BG model. The relationship between beta's (β) of the behavioral model and DP weights (w_Str→ GPi_) with a constant w_STN→GPi_ value (=0.75) used to attain **(A)** are shown in **(C)**. **(B)** Y axis represents percentage exploitation and X axis represents a subject which is a specific beta value (β) in behavioral model and the IP weight (w_STN→GPi_) in the BG model. The relationship between beta's (β) of the behavioral model and IP weights (w_STN→GPi_) of BG model with a constant w_Str→ GPi_ value of (=5) used to attain **(B)** are shown in **(D)**.

The performance of BG model was compared with the behavioral model in terms of % exploitation shown in the Figure 14. Figure 14A shows the % percentage exploitation as the Y-axis with x-axis as individual subjects, which relates to corresponding beta (β) values in behavioral and DP weight values in BG model. Holding w_STN→GPi_ constant at 0.75, we varied w_Str→ GPi_ over the range of [2, 4] in steps of 0.25 to match the exploitation levels of the subjects. The relationship between the DP weights (w_Str→GPi_,) and beta (β) is plotted in Figure 14C. Similarly % percentage exploitation for changing beta (β) and increasing (w_STN→GPi_) was plotted in Figure 14B. A decrease in w_Str→ GPi_ implies reduced influence of DP relative to IP, resulting in greater exploration. Similarly one can be control exploration by varying the strength of the IP (w_STN→GPi_). Holding w_Str→GPi_, constant at value (=5), we varied w_STN→GPi_ over the range of [0.25, 1.25]. The individual weight values for corresponding beta's have been plotted in Figure 14D.

To simulate the performance of PD subjects in the above model, we clamped the delta (δ) to a negative value (-20) (simulating low levels of DA) and checked the performance. We observed that the % exploitation decreased (=44%) compared to normals. The decrease in the performance of the PD off condition might be due to decreased exploration leading to the selection of suboptimal choice.

**Figure 15:**
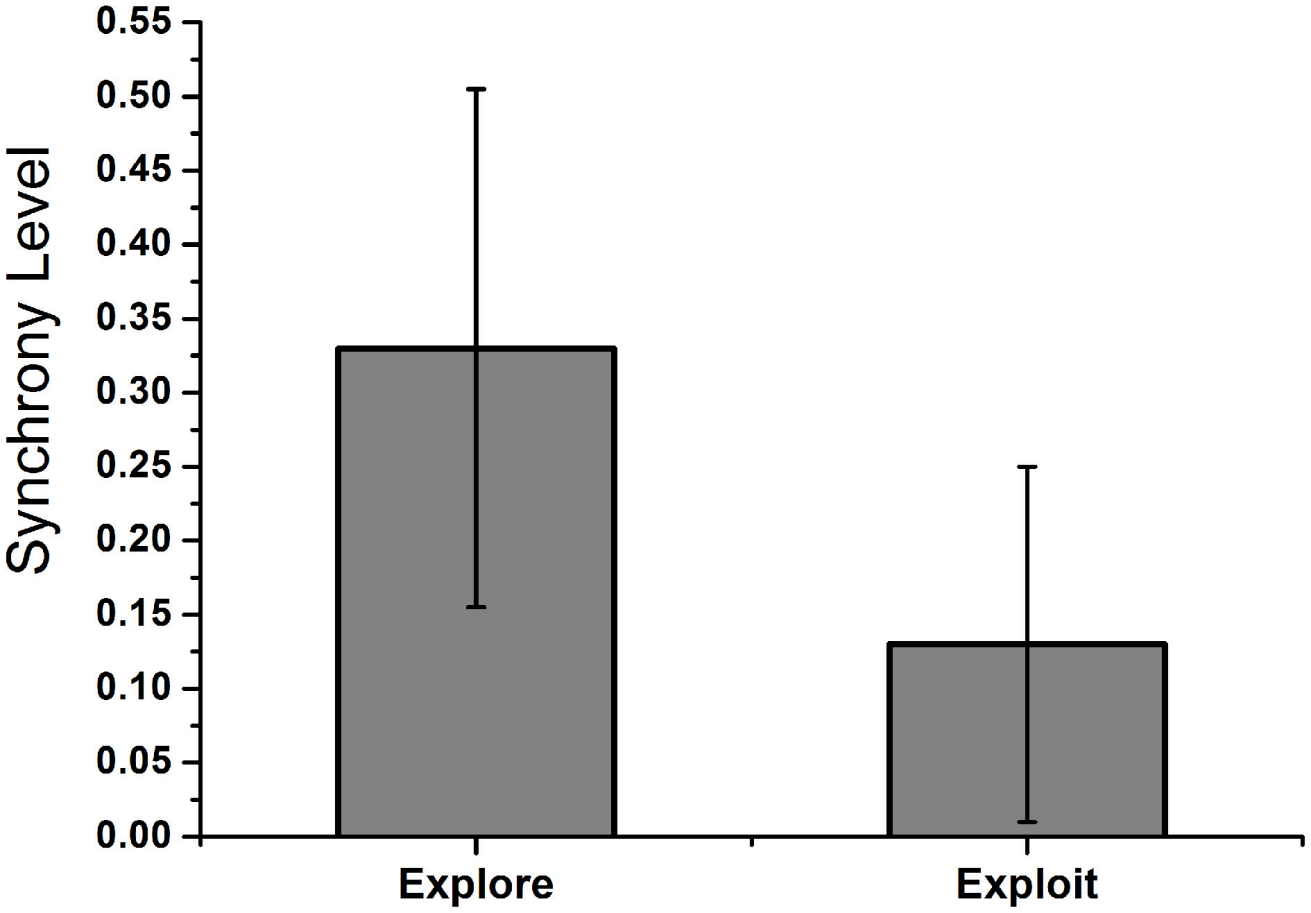
**Shows the mean “*****R***_***sync***_**” value of STN neurons during exploratory and exploitatory trials during the n-arm bandit task.** The trials were segregated in to exploitatory or exploratory and the corresponding “*R*_*sync*_” value of STN neurons was calculated. The exploitatory trials were high DA (0.7–0.9) levels where as exploratory ones around intermediate levels (0.4–0.6).

In the binary action selection task (Section Simulation Set 2: Binary Action Selection), we observed that the level of exploration could be related to the synchrony levels in STN neurons. So we classified each of the 300 trials into either exploratory or exploitatory and then checked the corresponding synchrony levels in STN neurons. The synchrony levels for exploitatory case was observed to be significantly lower (=0.13 ± 0.12) than exploratory ones (=0.33 ± 0.176). Independent 2 sample *t*-test was conducted between synchrony parameter “*R*_*sync*_” for exploratory and exploitatory trials. With a *P*-value of 0.002, we could say that there is a statistically significant difference between the 2 mean “*R*_*sync*_” values. The bar plot for mean “*R*_*sync*_” for explore and exploit trials is shown in Figure 15.

## Discussion

The goal of this model was to understand the role of the BG in explorative behavior as well as the occurrence of synchrony in PD conditions. Studies on exploration-exploitation tradeoff show the importance of these processes during decision making (Sutton and Barto, 1998; Cohen et al., 2007; Humphries et al., 2012; Laureiro-Martãnez et al., 2013). Experimental studies conducted by the Baunez group also suggest a role for STN in exploration where they observed that STN lesions tend to alter the choice made by rats (Baunez et al., 2001). These results emphasize the importance of studying exploration and the corresponding neural substrates at the subcortical level.

Exploration in action selection is usually modeled as being driven by noise or a stochastic mechanism (Cohen et al., 2007; Moustafa and Gluck, 2011; Schroll et al., 2012). The STN-GPe loop of BG has been proposed to act as a pacemaker (Plenz and Kital, 1999) capable of producing synchronized oscillations at low DA levels (PD) (Brown et al., 2001; Bevan et al., 2002) and desynchronized spiking activity at high DA level. In an earlier study, using a rate-coded neural network model of BG (Kalva et al., 2012), we have shown that the STN-GPe system exhibits chaos and fixed point dynamics as two network parameters (w = strength of connections between STN and GPe; σ = strength of lateral connections within STN and GPe) are varied. This trend reached its peak when the STN-GPe system was located on the border between chaos and ordered regimes, viz., the “edge of chaos.” From the facts that synchrony plays an important role in PD and DA levels influence the synchrony levels in STN-GPe, we developed a spiking neuron network model of BG to study the relation between synchrony and exploration by simulating simple (binary action selection) and complex decision making (n-armed bandit task). The model also showed oscillatory activity in STN-GPe neurons at low DA level known to correlate to PD tremor (Bevan et al., 2002).

### The STN-GPe loop and exploration-exploitation dynamics

One of the aims of the present study is to show that the complex dynamics of STN-GPe system contributes to exploration and can be correlated to the synchrony levels in the STN-GPe loop. So we studied the STN-GPe loop dynamics (Section Simulation Set 1: STN-GPe Circuit Dynamics and Synchrony) without input from D2 striatum for increasing levels of dopamine (Figures 5–7). As we are interested in studying the role of synchrony in exploration, we characterized the dynamics of STN-GPe in terms of synchronization and oscillatory activity (Figure 9). Due to the observations that STN and GPe neurons show synchrony (asynchrony) at low(high) DA levels (Bergman et al., 1994, 1998) and such behavior in excitatory-inhibitory networks is observed when the excitatory lateral connection is high and inhibitory is low (Lukasiewicz and Werblin, 1990). Considering the above observations we assume the DA modulates the width of Gaussians of STN and GPe collaterals. The results tally with the general observation from electrophysiology that at higher levels of dopamine, the STN-GPe system shows desynchronized activity and under dopamine-deficient conditions exhibits synchronized bursts (Bergman et al., 1994; Gillies and Willshaw, 1998; Park et al., 2011). It is also consistent with the experimental finding that dopamine-deficiency results in an increase of correlations in firing patterns of STN neurons (Brown et al., 2001; Benazzouz et al., 2002; Levy et al., 2002; Willshaw and Li, 2002; Brown, 2003; Foffani et al., 2005). Some computational modeling effort has investigated the link between STN, GPe oscillations and PD tremor (Bevan et al., 2002), an idea that also has strong experimental support with regard to the STN-GPe circuit (Nini et al., 1995; Hurtado et al., 1999; Levy et al., 2002; Brown, 2003; Park et al., 2010). We observed that STN activity showed oscillatory activity with a frequency (=10 Hz) which falls under the beta frequency range observed in experimental PD study (Weinberger and Dostrovsky, 2011).

### Role of STN-GPe in binary action selection task

We then used the same model to simulate the binary action selection task (similar to Humphries et al., 2006). Here, we presented two stimuli as inputs to the model (Figure 1). The firing rate of the stimulus was represented as its saliency (Humphries et al., 2006) where selection of higher one was defined as “exploitation/Go” and lesser one as “exploration/Explore” and not selecting any of the inputs as “No-Go.” In the BG model of Kalva et al. (2012) some action is always chosen—thus it does not have a “No-Go” regime (Kalva et al., 2012). The current Izhikevich BG network showed No-Go at low DA levels (0.1–0.3) and Go at high DA levels (0.7–0.9) consistent with the classical picture of BG function. Along with this a peak in “Explore” at intermediate levels of DA (0.4–0.6) was also observed (Figure 13). At intermediate levels of DA, the neuronal pools of STN corresponding to the 2 inputs were firing out of phase which was also observed in GPe and GPe neurons. This anti-phase spiking behavior of GPi neurons became the source of randomness in deciding which stimulus would finally get selected. In other words, the neuronal pool ahead in alternation crossed the threshold first and that corresponding action got selected. This exploratory behavior was controlled by the strength of laterals in STN neurons (A_STN_). As the strength of lateral connections was increased, the exploratory action selection percentage peaked and decreased on further increment (Figure B2 in Supplementary Material Appendix B). Increased A_STN_ leads to high synchrony among STN neurons which is one of characteristic feature observed in PD patients (Park et al., 2010, 2011). From the above observations we may suggest that the decrease in exploration levels in PD subjects (Archibald et al., 2013a) could be due to increased lateral strength in STN neurons value. We found at high lateral synaptic strength, the system switched only between Go and No-Go regimes (Figure B1a in Supplementary Material). To check whether any other module in the network is influencing exploration in the system, we removed the STN to GPi connection (which effectively eliminated the IP). This omission rendered the system to display only Go and No-Go regimes (no exploration) (Figure B1b in Supplementary Material). We also studied the effect of STN lesions on exploration and found as the size of lesion increased the system's exploratory behavior decreased. This result is in agreement with study conducted by Baunez et al. (2001) where they found a decrease in explorative behavior of rats while performing a choice reaction task (Baunez et al., 2001).

### Complex decision making: N-armed bandit task

In the n-arm bandit task, the aim of the subject was to maximize the reward by selecting best (highest reward giving) slot in each trial and their performance was measured in terms of amount of exploitative behavior. To make sense of the experimental data, a behavioral model was used to estimate which trial was explorative or exploitative. A similar model was used by Daw et al. (2006) to analyze their fMRI data (Daw et al., 2006). The behavioral model uses the classical soft-max principle (Equation A.8 Appendix A in Supplementary Material) and the parameter “β” controls the level of exploration. Though the behavioral model helps in analyzing the experimental data, it does not elaborate on underlying neural mechanism of exploration due to constraint of being abstract. Apart from understanding the neural mechanism for exploration, the model can also be used to study decision making ability in Parkinsonian conditions and predict the effect of various drugs (L-Dopa, DA agonist) and STN–DBS. We attempted to explain the decision making mechanism in terms of synchrony levels in STN-GPe neurons. When subjected to the binary action selection task, the spiking network model of BG showed exploration at intermediate levels of DA controlled by STN-GPe synchrony levels. So the amount of exploration in the model was controlled by adjusting the synaptic weights in DP (w_Str→ GPi_) and IP pathway (w_STN→GPi_).

The results of behavioral model of Bourdaud et al. (2008) were approximated in 2 ways: (1) Increasing w_STN→GPi_ while holding w_Str→ GPi_ constant; this would increase exploration since the the GPi neurons are now more strongly influenced by STN and (2) Decreasing w_Str→ GPi_ while keeping w_STN→GPi_ constant, which has the same effect of increasing w_STN→GPi_.

Two parameters (δ and % exploitation) were compared to check the accuracy of the model to account for experimental results. Individual performance of behavioral and BG models were checked by calculating delta (δ),TD error Equation (27) which indicates how well the model is able to track the actual reward pattern from the slots (Table 3). If the BG model is able to replicate the experimental results, the “δ” obtained from BG and behavioral (measure of experimental results) should be correlated. So we calculated the error *e*^*bebg*^ between the two delta's (δ^*bg*^ and δ^*be*^) to check the accuracy and found the error to be low (Table 3). We also conducted 2 sample *t*-test on the delta values obtained from BG and behavioral model. An “H” value of (=0) for the test cases indicates that both delta's are from distribution of equal means. In other words, the difference/error between the 2 model expected values is low. The second measure was percentage exploitation i.e., the percentage number of times the model selects the slot with the highest expected payoff (Figure 14). The results obtained from BG model closely match with the behavioral model reinforcing the theory that STN-GPe could be a source for exploration at sub-cortical level. The synchrony level in STN was also found to be statistically different (*P* = 0.002) during exploratory vs. exploitatory trials. The “*R*_*sync*_” value (Figure 15) during exploitatory trial (=0.13 ± 0.12) at high DA levels showed a desynchronized behavior leading to the selection of highest reward slot. During exploratory trials, synchrony level of (=0.33 ± 0.175) was observed in STN neurons which is similar to that as observed during binary action selection at intermediate DA level. This intermediate synchrony levels gave rise to the alternating pattern, source of randomness in the model leading to an exploratory behavior.

From these results, we would to emphasize that the exploratory behavior in the system can be controlled by collateral connection strength in STN neurons by changing the synchrony levels in the system. These results suggest that STN-GPe system of BG might be the possible exploratory substrate at subcortical level. Cortical structures also play a critical role in decision making (Bechara et al., 1994; Fellows and Farah, 2003; Clark et al., 2004; Ragozzino, 2007). Many research groups have been working to characterize the anatomical substrates of exploration and exploitation during decision making (Daw et al., 2006; Bourdaud et al., 2008; Pearson et al., 2009, 2010; Laureiro-Martãnez et al., 2013). The ability to modulate the oscillatory activity in STN-GPe neurons by cortex through the hyper-direct pathway has also been suggested and modeled (Kang and Lowery, 2013). Since most classical models of basal ganglia do not include the connection between GPe and GPi (Albin et al., 1989; Delong, 1990) but recent studies by (Nambu et al., 2005) indicate the presence of this pathway. Modeling studies such as (Coulthard et al., 2012) showed the role of BG in decision making without including this specific connection in their model. Though the presence of this pathway has been found out anatomically, the functional significance is yet to be explored. Considering these in to account, we have not included GPe-GPi connection in the model. As a part of future work we would like to integrate cortical areas and the inhibitory GPe-GPi connection with the current model and study the rich dynamics in the system.

### Conflict of interest statement

The authors declare that the research was conducted in the absence of any commercial or financial relationships that could be construed as a potential conflict of interest.
