# Supplementary material for: A spiking Basal Ganglia model of synchrony, exploration and decision making
Source: Front Neurosci. 2015 May 27;9:191. doi: 10.3389/fnins.2015.00191 (PMC4444758; doi:10.3389/fnins.2015.00191)
Supplement: Supplementary file 1 [file DataSheet1.DOCX]

**Appendix A**

Behavioral model (adapted from Bourdaud et al. (2008)).

The behavioral model labels each trial as corresponding to either an exploratory or exploitative decision. The model assumes that the user estimates the mean payoff of each machine using a Bayesian linear Gaussian rule (i.e., a Kalman filter). Using these estimations, he/she selects a machine according to a softmax rule. All the subjects are assumed to share the same model for tracking the payoff means and thus parameters are computed using the entire available data. The parameters of the model (for both mean tracking and machine selection) are estimated by maximizing the model likelihood with respect to the subject’s choices.

At any given trial, the behavioral model provides the mean payoff for all machines considering previous observations (i.e., the payoff obtained at previous trials). Comparison between the model’s estimated payoffs for all machines is used to label that trial as either exploration or exploitation. Those trials in which the user selects the machine with the highest estimated mean are labeled as corresponding to exploitative decisions.

The subject strategy for tracking the payoff of each machine is modeled by a Kalman filter, whose parameters are assumed to remain constant over trials. Once the j^th^ machine is selected, at the k^th^ trial, the estimated payoff distribution is updated from its pre-selection values ($\widehat{\mu}_{j,k}^{pre},(\widehat{\sigma}_{j,k}^{pre})^{2}$) to its post-selection values ($\widehat{\mu}_{j,k}^{post},(\widehat{\sigma}_{j,k}^{post})^{2}$) as follows

$\widehat{\mu}_{j,k}^{post}=\widehat{\mu}_{j,k}^{post}+K_{k}(r_{k}-\widehat{\mu}_{j,k}^{pre})$ (A.1)

$(\widehat{\sigma}_{j,k}^{post})^{2}=(1-K_{k})(\widehat{\sigma}_{j,k}^{pre})^{2}$ (A.2)

where

$(K_{k})=\frac{(\widehat{\sigma}_{j,k}^{pre})^{2}}{(\widehat{\sigma}_{j,k}^{pre})^{2}+(\widehat{\sigma}_{0})^{2}}$ (A.3)

The mean estimation for the remaining machines does not change as result of the choice since the user cannot observe the payoff of these machines. That is,

$$\forall i\neq j$$

$\widehat{\mu}_{j,k}^{post}=\widehat{\mu}_{j,k}^{pre}$ (A.4)

$\widehat{\sigma}_{j,k}^{post}=\widehat{\sigma}_{j,k}^{pre}$ (A.5)

Then, the estimations are also evolved according to the diffusion rule:

$\widehat{\mu}_{j,k+1}^{pre}=\widehat{\lambda}\widehat{\mu}_{j,k}^{post}+(1-\widehat{\lambda})\widehat{\theta}$ (A.6)

(A.7)

The choice of subjects is modeled by a softmax rule, i.e., at each trial k the probability of choosing the machine is

$P_{i,k}=\frac{\exp(\beta\widehat{\mu}_{i,k}^{pre})}{\sum_{j} \exp(\beta\widehat{\mu}_{j,k}^{pre})}$ (A.8)

Where ‘β’ is a scaling parameter. Higher values of β drive the system to exploitative behavior and vice versa. The parameters of the behavioral model ($(\sigma_{0},\widehat{\theta},\widehat{\lambda},\widehat{\sigma}_{d})$are estimated by maximizing the log likelihood under the following constraints. To speed up convergence, estimated parameters are initialized to the parameters of the original modelrespectively. Fixing the last two parameters does not significantly affect the estimation of the others, because their influence vanishes quickly within a few trials. Table A.1 shows the estimated values of the model, which are consistent with the real values of the machines.


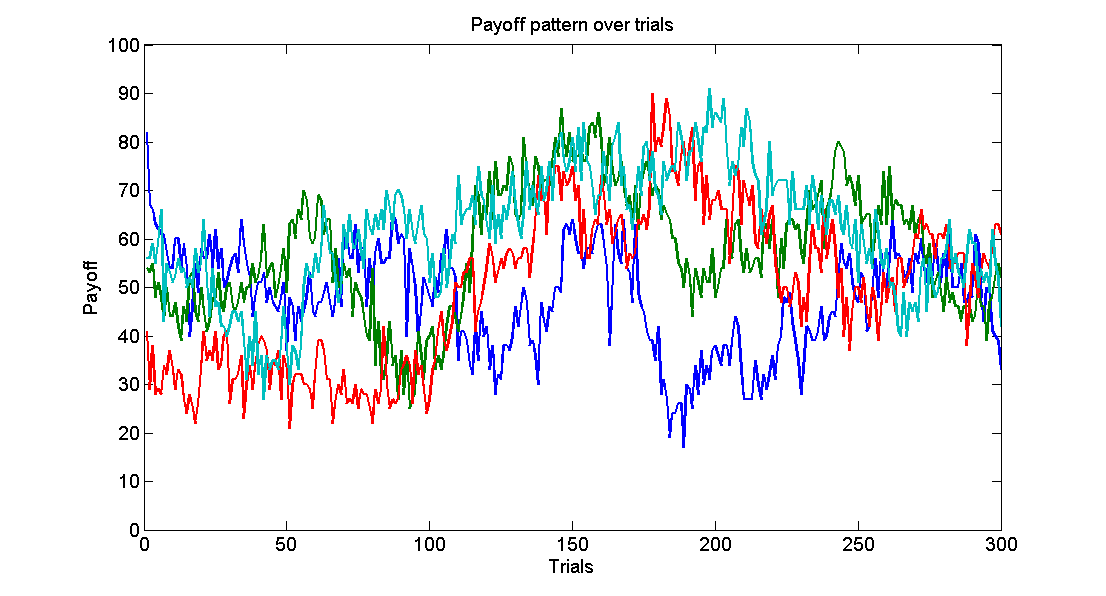


Figure A1 shows a sample payoff obtained from the 4 bandits for 300 trials represented by 4 colors. The reward for each trial was calculated using eqn. (19 & 21)

**Table A.1**

Estimation of parameters of the behavioral model (Bourdaud et al., 2008)

|  | λ | θ | σ_d_ | σ_0_ |
| --- | --- | --- | --- | --- |
| Real Values  Estimated Values | **0.9836**  **0.92** | **50**  **51.37** | **2.8**  **8.12** | **4**  **N/A** |
| Subject  β | **1 2**  **0.37 0.28** | **3 4**  **0.19 0.21** | **5 6**  **0.19 0.29** | **7 8**  **0.29 0.23** |

**Appendix B**


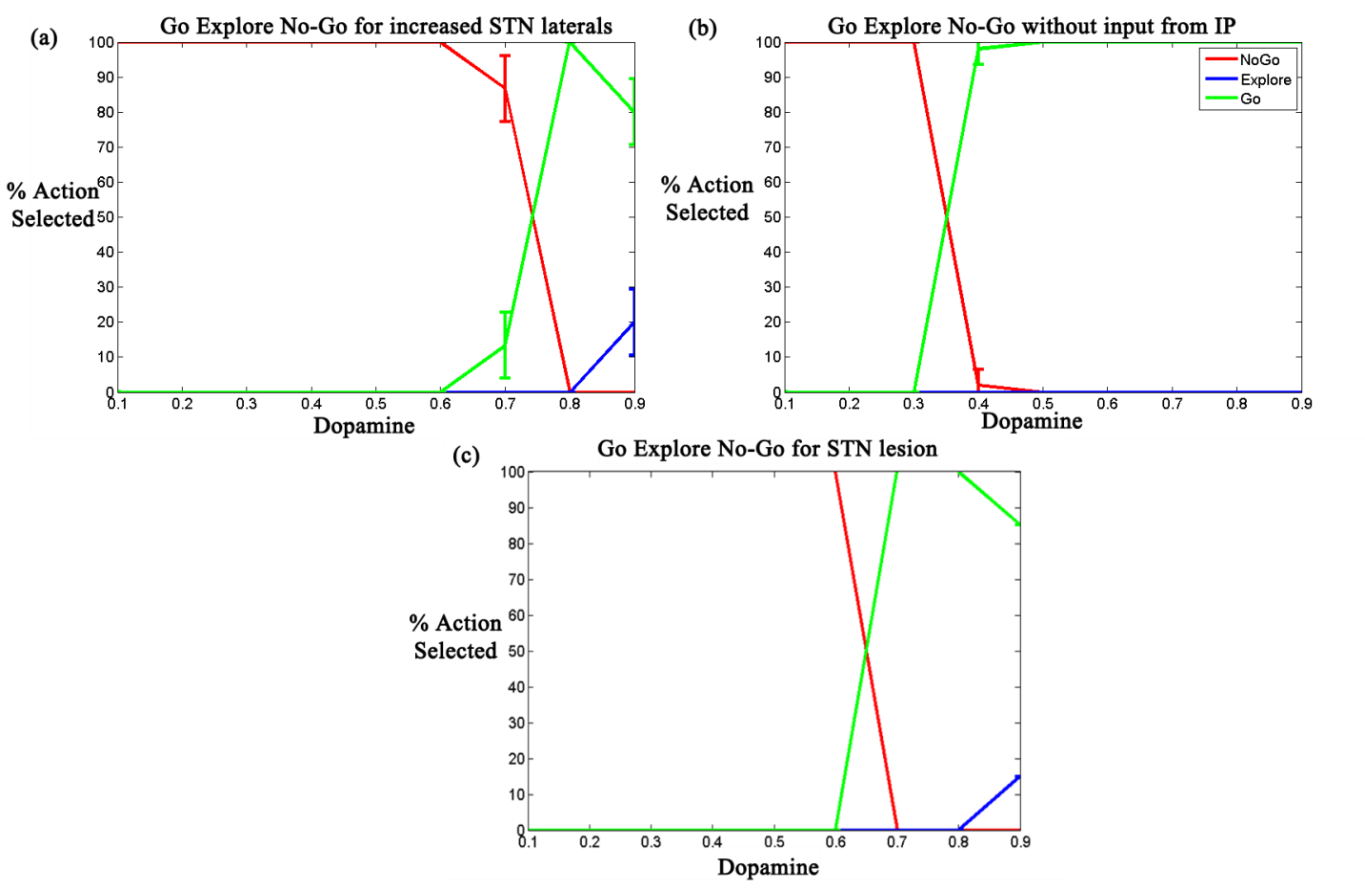


Figure B1: The Go Explore No-Go (GEN) regime for three different cases, to emphasize the importance of lateral connections in the STN-GPe network. (a)The lateral synaptic weights in STN were increased (A_STN_ = 0.3) and exploration levels dropped (b) The inputs from STN to GPi were removed and the system displaced only No-Go and Go profiles (c) Lesioning of a patch of STN neurons(20x20) patch at the center of the lattice, there is hardly any exploration present in the system.


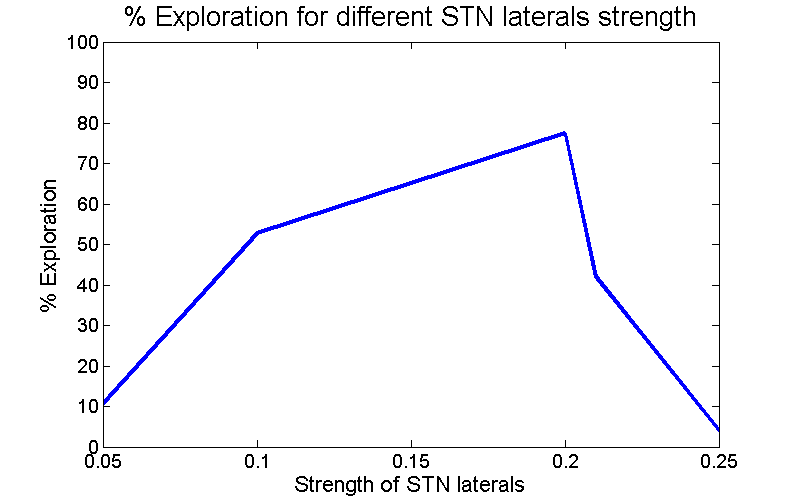


Figure B2. The relationship between exploration and strength of STN laterals. Figure shows the % exploration level as the strength of the STN laterals is increased. The exploration is high for values within a range of [0.1 0.2] and is found be less for low & high values.
